# Improvement of Osteogenic Differentiation of Mouse Pre-Osteoblastic MC3T3-E1 Cells on Core–Shell Polylactic Acid/Chitosan Electrospun Scaffolds for Bone Defect Repair

**DOI:** 10.3390/ijms25052507

**Published:** 2024-02-21

**Authors:** Francesco Lopresti, Simona Campora, Salvatrice Rigogliuso, Aldo Nicosia, Alessandra Lo Cicero, Chiara Di Marco, Salvatore Tornabene, Giulio Ghersi, Vincenzo La Carrubba

**Affiliations:** 1 Department of Engineering, University of Palermo, RU INSTM, Viale delle Scienze, 90128 Palermo, Italy; chiara.dimarco02@unipa.it (C.D.M.); salvatore.tornabene@community.unipa.it (S.T.); vincenzo.lacarrubba@unipa.it (V.L.C.); 2 Department of Biological, Chemical and Pharmaceutical Sciences and Technologies (STEBICEF), University of Palermo, Viale delle Scienze, Ed. 16, 90128 Palermo, Italy; salvatrice.rigogliuso@unipa.it (S.R.); alessandra.locicero@unipa.it (A.L.C.); giulio.ghersi@unipa.it (G.G.); 3 Institute for Biomedical Research and Innovation, Italian National Research Council (IRIB-CNR), 90146 Palermo, Italy; aldo.nicosia@irib.cnr.it; 4 Abiel s.r.l, via Enzo ed Elvira Sellerio, 50, 90141 Palermo, Italy; 5 ATeN Center, University of Palermo, Viale delle Scienze, Ed. 18A, 90128 Palermo, Italy

**Keywords:** polylactic acid, chitosan, cold plasma treatment, osteoblasts, cells differentiation, gene expression

## Abstract

Electrospun hybrid scaffolds composed of synthetic and natural polymers have gained increasing interest in tissue engineering applications over the last decade. In this work, scaffolds composed of polylactic acid electrospun fibers, either treated (P-PLA) or non-treated (PLA) with air-plasma, were coated with high molecular weight chitosan to create a core–shell microfibrous structure. The effective thickness control of the chitosan layer was confirmed by gravimetric, spectroscopic (FTIR-ATR) and morphological (SEM) investigations. The chitosan coating increased the fiber diameter of the microfibrous scaffolds while the tensile mechanical tests, conducted in dry and wet environments, showed a reinforcing action of the coating layer on the scaffolds, in particular when deposited on P-PLA samples. The stability of the Chi coating on both PLA and P-PLA substrates was confirmed by gravimetric analysis, while their mineralization capacity was evaluated though scanning electron microscopy (SEM) and energy-dispersive spectroscopy (EDS) after immersing the scaffolds in simulated body fluids (SBF) at 37 °C for 1 week. Sample biocompatibility was investigated through cell viability assay and SEM analysis on mouse pre-osteoblastic MC3T3-E1 cells grown on scaffolds at different times (1, 7, 14 and 21 days). Finally, Alizarin Red assay and qPCR analysis suggested that the combination of plasma treatment and chitosan coating on PLA electrospun scaffolds influences the osteoblastic differentiation of MC3T3-E1 cells, thus demonstrating the great potential of P-PLA/chitosan hybrid scaffolds for bone tissue engineering applications.

## 1. Introduction

Aiming at the fabrication of advanced biopolymeric porous structures for regenerative medicine purposes, hybrid scaffolds form a direction of research in the pursuit of suitable implants [1,2,3,4]. Hybrid scaffolds can be composed of synthetic and natural biopolymers, thus potentially exhibiting the advantages of both kinds of materials e.g., high mechanical properties and the ability to support cell attachment and proliferation [5,6]. Among the diverse approaches proposed for scaffold fabrication, electrospinning is gaining increasing interest due to its versatility and simplicity [7,8,9,10]. Furthermore, electrospinning approaches are suitable for the fabrication of hybrid scaffolds by blending or geometrically hierarchizing synthetic and natural biopolymers [11,12]. Blended hybrid scaffolds usually exhibit intermediate mechanical properties and the ability to support cell attachment and proliferation depending on the type of synthetic and natural biopolymers involved and their relative ratios [13]. Meanwhile, electrospun fibers structured in core–shell architectures offer the highest potential in exhibiting the advantages of synthetic and natural materials involved [14,15]. Electrospun core–shell scaffolds can be obtained in different ways, although the most common is the use of coaxial needles or of emulsion electrospinning [16]. The first method involves the use of two different polymeric solutions to be individually injected into the inner or outer needle of the coaxial system [17]. The second approach relies on the formation of a stable emulsion of two polymeric phases to be released by a single needle for the processing [18]. Both systems are characterized by specific advantages and disadvantages, although both require a complicated set of processing parameters related to the different properties of the two phases involved. Recently, the suitability of a direct coating approach has been demonstrated for the simple and reproducible fabrication of core–shell microfibrous scaffolds composed of synthetic (core) and natural (shell) polymers [1].

For bone regenerative purposes, polylactic acid (PLA) is considered one of the most adequate synthetic polymers due to its interesting properties. PLA is an FDA-approved linear aliphatic polyester that exhibits suitable biocompatibility, mechanical characteristics, and degradability for bone tissue engineering applications [19,20]. However, several studies have highlighted doubts regarding the poor hydrophilicity of PLA, which might inhibit its biocompatibility in terms of the amount of absorbed proteins and cell adhesion [21].

Chitosan (Chi), a chitin derivative, has been widely explored as a natural biopolymer suitable for bone regeneration, thanks to its biocompatibility, biodegradability, and intrinsic antibacterial nature [22,23]. Chitosan has a polymeric structure similar to glucosamine, which improves cell adhesion, proliferation, and differentiation [24] and eases hydroxyapatite formation in an osteogenetic environment [25,26]. Therefore, it is involved in mineralization during osteoblast differentiation by regulating osteoblastic genes [27,28]. However, chitosan-based constructs can lack mechanical stability or might offer low elastic modulus and tensile strength to be used for direct implantation [29]. Therefore, several hybrid scaffolds composed of PLA and chitosan have been prepared for skin [30,31,32,33], ligament [34], and bone [35,36,37,38] tissue engineering applications. However, to the best of our knowledge, examples of PLA/Chi core–shell electrospun scaffolds for bone tissue engineering applications have yet to be reported. In recent years, cold plasma technology has emerged as an effective alternative to traditional chemical methods for the enhancement of the adhesion properties of polymeric substrates with natural molecules [39].

In this work, core–shell PLA/Chi microfibrous hybrid scaffolds were produced by the direct coating of Chi solution on prepared PLA scaffolds with and without pretreatment with air-cold plasma (P-PLA).

The hybrid scaffolds were prepared by a direct coating of PLA or P-PLA electrospun fibers with chitosan solubilized, at different concentrations, in a water/acetic acid solution to evaluate the possibility of tuning the Chi coating thickness. The Chi coating affected the fibers morphology, mechanical properties, wettability, and mineralization when immersed in SBF. Furthermore, the plasma pretreatment on PLA influenced the chitosan coating morphology and its stability in water. Finally, biocompatibility of each type of scaffold was investigated by seeding pre-osteoblastic MC3T3-E1 cells and the effects of chitosan and plasma treatment on cell proliferation, differentiation and gene expression were evaluated by biological assays.

## 2. Results and Discussion

### 2.1. Coating Efficacy, Morphology, and Wettability of Scaffolds

The effect of the plasma treatment on the PLA electrospun scaffolds was evaluated by means of XPS analysis. Table 1 shows the atomic percentages determined from the areas of the C 1s and O 1s peaks and the fraction of carbon functional groups from high resolution C 1s XPS peaks. The results reveal that the air plasma treatment increased the overall oxygen content at the surface by approximately 2 atomic% units thus increasing the O/C ratio from 0.54 to 0.60. Furthermore, the saturated hydrocarbon C–C C 1s peak decreased after plasma treatment, with a concomitant increase in the intensities of C–O (from 25.98% to 29.74%) and O=C–O (from 24.81% to 30.12%) peaks. This can be attributed to the introduction of oxygen functionalities and to the increase in carboxyl and hydroxyl groups on the surface, as already observed in a previous work [40].

**Figure 1:**
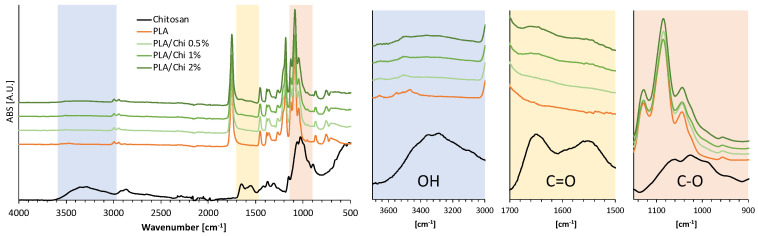
FTIR–ATR spectra of PLA/Chi hybrid scaffolds at different chitosan concentrations in the coating solutions. The functional groups reported in the insets refer to the chitosan spectra.

The presence of chitosan molecules on the PLA electrospun microfibers was evaluated via FTIR-ATR spectroscopy (Figure 1). For the sake of brevity, FTIR-ATR spectra of P-PLA/Chi samples are not shown, as no significant differences were found in comparison with PLA/Chi results. According to the literature, the ATR-FTIR spectrum of PLA showed various absorption bands usually attributed to this polyester, such as the carbonyl stretch at 1747 cm^−1^; the C–O stretch at 1180 cm^−1^, 1129 cm^−1^ and 1083 cm^−1^; and the OH bend at 1044 cm^−1^ [41]. The FTIR-ATR spectrum of neat chitosan powder reveals main absorption bands at 3352 cm (stretching vibration of O–H group) [42], 3290 cm^−1^ (stretching vibration of N–H) [42], 1645 cm^−1^ (stretching vibration of the carbonyl group (C=O) in amide I) [43,44], and 1039 cm^−1^ (stretch vibrations of C–O) [45].

The peaks related to PLA in the PLA/Chi hybrid scaffolds were found to be dominant; however, the characteristic peaks of chitosan were visible, and their intensity increased with increasing chitosan concentration, thus corroborating the effective inclusion of the polysaccharide into the PLA electrospun scaffolds (Figure 1). Furthermore, no evident shifts of either PLA, or Chi characteristic peaks were detected, thus suggesting that no chemical interaction occurred between the two phases. 

**Figure 2:**
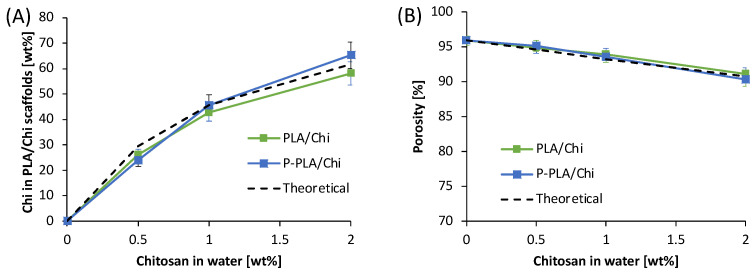
(**A**) Chitosan concentration and (**B**) porosity of PLA/Chi hybrid scaffolds as a function of the chitosan concentration in the coating solutions.

Weight changes of PLA/Chi scaffolds after chitosan deposition also validated the hypothesis of successful integration of chitosan into the hybrid scaffolds. The weight concentration of Chi in the PLA/Chi scaffolds was measured according to Equation (3) and compared with the theoretical values, as shown in Figure 2A. The measured weight changes of the hybrid scaffolds after Chi inclusion were quite similar to the theoretical ones, suggesting the fine control of the weight composition of the final hybrid scaffolds. Changes in the porosity of the scaffolds after chitosan inclusion are reported in Figure 2B. Results reveal that, as expected, the porosity values were slightly affected by the coating, as they decreased from 96% of PLA scaffolds to the lowest value of around 90% for PLA/Chi 2% and P-PLA/Chi 2% samples. The theoretical values of porosity, shown in Figure 2B, were obtained by assuming that the theoretical amount of chitosan fills the void volumes of the PLA scaffold, adopting the mean porosity of the PLA scaffolds as starting value. Additionally, the porosity values, calculated according to Equation (1), were superimposed to the theoretical values, as the chitosan addition to PLA electrospun scaffolds have not changed the sizes of the samples while modifying their weight and the matrix density. Furthermore, the porosity values were affected by the plasma pretreatment, but only by the concentration of chitosan solubilized in the coating solution. This result suggests the hypothesis that the mechanism involved in the coating formation, related to the void filling, is not dependent on the interaction occurring between the polymeric surface and the coating solution.

**Figure 3:**
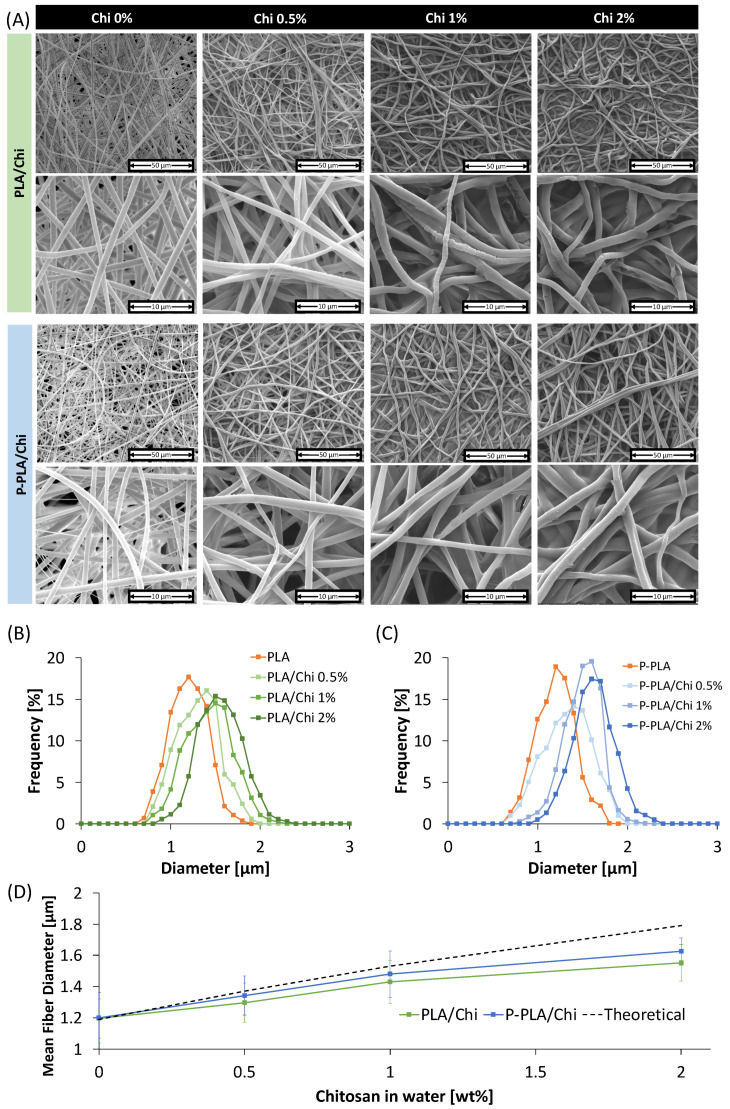
(**A**) SEM micrographs of PLA/Chi hybrid scaffolds at different chitosan concentrations with and without plasma pretreatment; diameter size distribution of (**B**) PLA/Chi scaffolds and (**C**) P-PLA/Chi scaffolds; and (**D**) mean fiber diameter of the electrospun fibers as a function of chitosan concentration, compared with the theoretical values.

The microstructure of the scaffold was investigated via SEM analysis and the results are shown in Figure 3A while, in Figure 3B,C, the fiber diameter distribution of PLA/Chi and P-PLA/Chi scaffolds are reported, respectively. PLA and P-PLA fibers displayed almost identical fiber morphology with smooth fibers, randomly oriented and with mean diameters of around 1.2 µm. 

These results highlight how the air plasma process did not remarkably affect the PLA fiber morphology, which is consistent with a previous work [1]. The addition of chitosan caused several modifications to the morphology of the PLA and P-PLA fibers. Firstly, in Figure 3A, one can clearly notice an increase of the fiber diameter with increasing chitosan content. This result was confirmed by the fiber diameter distribution and the value of the mean fiber diameter evaluated via image processing software (Figure 3B–D). Interestingly, the mean fiber diameters of the PLA/Chi 0.5% and P-PLA/Chi 0.5% scaffolds were very close to the expected values. As the theoretical values of the fiber diameters were obtained by assuming that the whole volume of added chitosan increased the starting mean diameter of PLA and P-PLA fibers, this result demonstrated that, at this concentration, chitosan film effectively grows around individual PLA and P-PLA fibers during the water evaporation, thus wrapping them and assembling a core–shell configuration.

The fiber diameters of PLA/Chi and P-PLA/Chi scaffolds treated with more concentrated chitosan solutions were higher than those of PLA/Chi 0.5% and P-PLA/Chi 0.5% but lower than the theoretical values, in particular for the PLA/Chi 2% and P-PLA/Chi 2% hybrid scaffolds (Figure 3D). 

This result can likely be explained by observing the SEM images at higher magnification in Figure 3A. The images reveal edges among the fibers of PLA/Chi and P-PLA/Chi 1% and 2%, which can presumably be ascribed to an excess of chitosan that is unable to wrap the PLA fibers during the solidification and thus unable to increase the PLA and P-PLA fiber diameter to the theoretical values. Furthermore, for those scaffolds, bundles composed of two to four fibers glued to each other can be observed. The only relevant morphological difference observed between PLA/Chi and P-PLA/Chi hybrid scaffolds concerns their fiber roughness, independently from the chitosan solution used. In fact, from SEM images at higher magnification, it is possible to note that the PLA/Chi fibers displayed an accordion-like structure, significantly evident for PLA Chi 1%.

This result was already observed in a previous work in relation to the wettability differences of PLA and P-PLA fibers [1]. In fact, the shape of the chitosan coating on the PLA and P-PLA fibers reflects the shape of the chitosan solution during the water evaporation. It can be assumed that, on hydrophobic PLA fibers, the film solution is not always continuous but forms a series of adjacent droplets, resulting in the accordion structures noted in the PLA/Chi fibers. Meanwhile, the chitosan solution is able to wet the hydrophilic P-PLA fibers easily, resulting in the smoother fiber morphology of the P-PLA/Chi hybrid scaffolds.

Furthermore, EDS analysis (Table 2) revealed that the elemental composition of the scaffold’s surface is moderately affected by the plasma treatment and is strongly affected by the presence of the chitosan coating. 

In more detail, the plasma treatment induced a slight increment of the elemental concentration of oxygen on the scaffold’s surface from 82.9 to 83.3 wt%. 

As expected, the presence of chitosan was revealed by the presence of a peak related to N atoms. Interestingly, the weight concentration of N atoms slightly increased with increasing chitosan concentration in the coating solution. The N atoms revealed by EDS can be related to the amide groups present in the chitosan molecule and detected by the FTIR-ATR analysis. 

**Figure 4:**
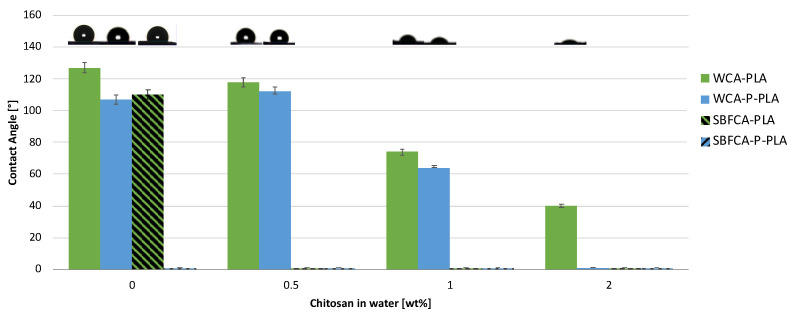
WCA and SBFCA values, evaluated on PLA/Chi P-PLA/Chi hybrid scaffolds.

Scaffold wettability is a key parameter as it affects cellular behavior in terms of adhesion and its ability to be permeated by culture medium [46]. CA contact angle measurements conducted using water (WCA) or SBF (SBFCA) as liquid are reported in Figure 4. Results show that both WCA and SBFCA values decreased with increasing concentration of chitosan in the coating solutions. In further detail, WCA of PLA/Chi systems decreased from 126° ± 5° for PLA to 118° ± 3° for PLA/Chi 0.5% and then linearly decreased to 40° ± 1° for PLA/Chi 2%. A similar trend was observed for P-PLA WCA values, one that was comparable to P-PLA and P-PLA/Chi 0.5% (about 110°) and then linearly decreased to 0° with increasing concentration of chitosan in the coating solution. 

SBFCA was found to be different from 0° only when the test was performed on the PLA sample. In general, results reveal an increment of the scaffold’s hydrophilicity with an increasing concentration of chitosan in the coating solution. These results were highly expected, as the wettability performance of electrospun scaffolds is strongly dependent on both the surface chemical properties of the materials and on its topography [47]. The high hydrophobicity of electrospun PLA can be attributed to the influence of surface texture, as already reported in previous studies [48]. Air plasma treatment is well known to form oxygenated moieties due to two main mechanisms: oxidation and the molecular destruction of PLA [49], confirmed in this work by the EDS analysis. The oxygenated functional groups are able to increase the interfacial tensions at the solid–liquid interface, resulting in a decrease of the WCA [50]. Similarly, the Chi coating produced the same results, which can be ascribed to its hydrophilic chemical structure, resulting in the presence of a higher number of hydroxyl groups on the surface of the coated samples, as recorded by FTIR-ATR measurements [51]. Interestingly, SBFCA values were found to be consistently lower than those of WCA, as a rapid SBF droplet absorption was observed, except for the PLA scaffold. This result was likely driven by the capillary forces induced by the high porous structure of the scaffolds. As the scientific literature has reported that the interfacial tensions of the solid–vapor interface for water and SBF are similar (about 72 mJ m^−2^) [52], it can be supposed that SBF has a higher affinity to the produced substrates than water. 

### 2.2. Mechanical Properties of Hybrid Scaffolds

The mechanical properties of the scaffolds were evaluated in dry condition and with samples immersed in PBS at 37 °C (wet condition) in order to simulate their real working conditions.

**Figure 5:**
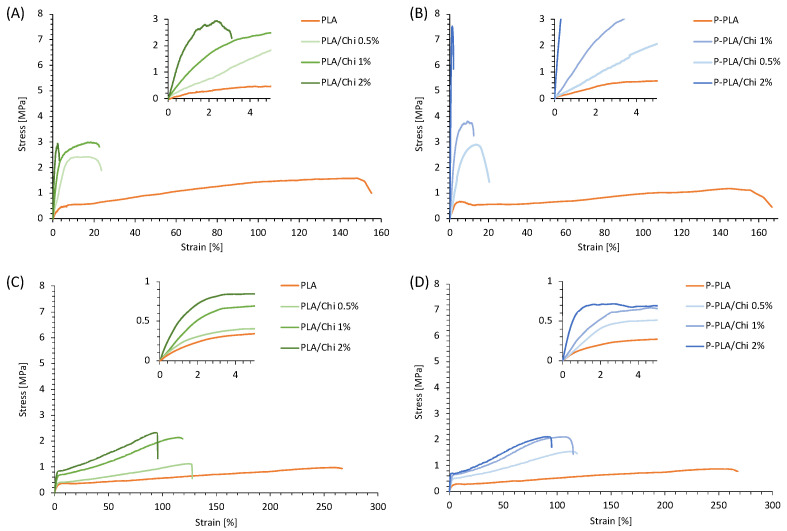
Representative stress–strain curves, recorded in a dry environment, of (**A**) PLA/Chi and (**B**) P-PLA/Chi hybrid scaffolds. Representative stress–strain curves, recorded in PBS at 37 °C, of (**C**) PLA/Chi and (**D**) P-PLA/Chi hybrid scaffolds.

In Figure 5A,B are reported the representative stress–strain curves of PLA and P-PLA based scaffolds, respectively, as obtained from tests conducted in dry conditions. Figure 5C,D report the same curves recorded in wet conditions. The curves display the way in which PLA and P-PLA scaffolds exhibit a behavior characterized by a relatively wide plastic deformation region in both dry and wet conditions. In dry conditions, the hybrid scaffolds are dramatically more brittle than PLA and P-PLA scaffolds and they exhibited higher elastic modulus (E) and tensile strength (TS). The inserts in Figure 5A,B make evident the marked slope increase of the linear elastic region of the stress–strain curves with an increase in concentration of the coating chitosan solution. This result is also visible in wet conditions (Figure 5C,D), although the differences in terms of elastic modulus and elongation at break among the hybrid scaffolds and the uncoated samples are less remarkable. 

Table 3 summarizes the E, TS and deformation at break (ε_b_) evaluated by the nominal stress–strain curves. In dry conditions, the elastic modulus of PLA/Chi scaffolds displayed a dramatic increase when coated with chitosan-based solutions and this increased with an increase in the chitosan concentration, reaching values more than 10 times higher than PLA scaffolds. When comparing the hybrid scaffolds with the same chitosan concentration, the P-PLA/Chi elastic modulus increment was higher than that of the PLA/Chi and this difference was more pronounced with increasing amounts of Chi.

Additionally, the TS of PLA/Chi scaffolds recorded in dry conditions were higher than those of PLA, though the increase was visible up to PLA/Chi 0.5% and then remained constant for the PLA/Chi 1% and 2% hybrid scaffolds. In contrast, TS values of P-PLA/Chi hybrid scaffolds increased almost linearly with increasing chitosan content for all of the investigated chitosan coating concentrations. As can be observed by the stress–strain curves, the deformation at break was dramatically reduced by the addition of chitosan to the PLA and P-PLA scaffolds. In this case, the decrease of this parameter was higher for the P-PLA/Chi scaffolds when compared with the PLA/Chi systems treated with the same chitosan solution concentration. 

In wet conditions, with respect to PLA scaffolds, the elastic modulus of the PLA/Chi scaffolds displayed a slighter, though still significant, increase when coated with chitosan than in the dry conditions, reaching maximum Chi concentration values that were more than three times higher than those of the PLA scaffolds. The effect of the plasma pretreatment on the elastic modulus of the samples in wet conditions is less effective, as a significant increase was recorded only for P-PLA/Chi 1% and 2% when compared with PLA/Chi 1% and 2%. Similarly, TS recorded in wet conditions were found to be consistently lower than in dry conditions and are more uniform among the different samples. Interestingly, by comparing each sample, one can see that elongation at break, when evaluated in wet conditions, are consistently higher than that in dry conditions. In fact, PLA/Chi and P-PLA/Chi samples showed a decrease in the elongation at break with an increase in the concentration of chitosan in the solution; however, the values also remained higher than 90% at the highest Chi concentration. 

The mechanical properties of the prepared hybrid scaffolds can be ascribed to several characteristics including (i) porosity, (ii) fiber morphology, and (iii) affinity between PLA (or P-PLA) and chitosan. 

In general, the increase of the elastic modulus of the hybrid scaffolds with respect to the PLA and P-PLA scaffolds agrees with previous work and can be ascribed to the gluing effect of the chitosan coating on the electrospun fibers [1]. In fact, the Chi coating may hinder the slipping of the fibers during the uniaxial tensile test, resulting in an increase of the elastic modulus. The reduction of the elongation at break of the hybrid samples corroborates this hypothesis. In fact, when fibers are free to move and slide over each other, high values of deformation are recorded. On the other hand, when fibers are glued by chitosan, their fracture occurs at a lower deformation degree.

Despite these general considerations, evident differences in the mechanical properties between PLA/Chi and P-PLA/Chi scaffolds were recorded, in particular when tested in dry conditions. An analysis of porosity highlighted the way in which Chi coating lowers the porosity of the scaffolds as a function of the Chi wt% in water, but that this parameter was not affected by the plasma pretreatment of the PLA scaffolds. As a result, the porosity cannot explain the elastic modulus differences observed between PLA/Chi and P-PLA/Chi systems. The fiber morphology of the P-PLA/Chi fibers was smoother and showed a more homogenous diameter size distribution, although the mean fiber diameter of the PLA/Chi and P-PLA/Chi scaffolds were close to each other. However, the presence of the accordion-like irregularities observed in the PLA/Chi fibers can act as defects of the structures, thus globally weakening the scaffolds. 

The distinctive tensile properties of PLA/Chi and P-PLA/Chi scaffolds can also likely be ascribed to the different affinity between PLA or P-PLA, and chitosan. According to the scientific literature [53], the adhesive forces between chitosan and the polymeric substrate can be ascribed mainly to the hydrogen bonding occurring between the oxygenated functional groups of PLA and hydroxyl groups of chitosan observed via FTIR-ATR analysis. It is well known that air–plasma treatments increase the oxygenated functional groups on PLA substrates [49,54], thus explaining the higher affinity of chitosan to P-PLA rather than to PLA and the resulting improvement of the mechanical properties.

The significant differences observed in the mechanical properties of the scaffolds when analyzed in dry and in wet conditions may be mainly ascribed to an interaction of water molecules with the Chi coating but can also be ascribed to the testing temperature. In fact, the reduction of E and TS and the increase of the elongation at break of the samples tested in wet conditions may be related to the ability of water to reduce the interaction among the Chi molecules in the contact regions of overlapped fibers, resulting in a reduction of the gluing effect of the chitosan coating on the electrospun fibers. Furthermore, the wet test was carried out at 37 °C, while the dry test was carried out at ambient temperature (23 °C). The increase of the temperature can increase the mobility of the polymeric chains in coherence with the mechanical properties recorded. Other phenomena that can likely explain the results include a swelling of the chitosan coating due to the inclusion of water molecules and the release of a slight amount of chitosan, as will be discussed below. Other phenomena, such as the swelling of PLA or the degradation of the matrix, may be excluded, as the samples were conditioned in PBS for only 30 min before the analysis. 

### 2.3. Thermal Properties of the Scaffolds

**Figure 6:**
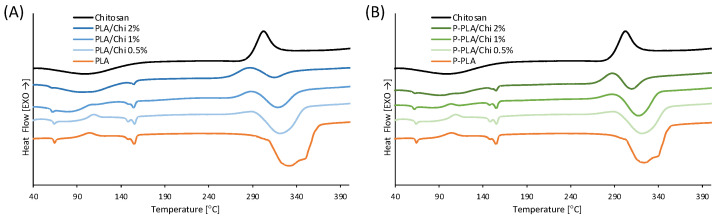
DSC thermograms of (**A**) Chi, PLA, and PLA/Chi scaffolds and (**B**) Chi, P-PLA, P-PLA/Chi scaffolds.

Differential scanning calorimetry (DSC) was carried out to evaluate the thermal properties of the PLA/Chi and P-PLA/Chi scaffolds as functions of Chi concentration. The thermograms are reported in Figure 6 while Table 4 summarizes the main thermal parameters obtained from the curves.

PLA and P-PLA thermograms are quite similar and each highlighted a first endothermic peak at around 64 °C, due to the glass transition temperature. PLA and P-PLA also showed a second exothermic peak at 102 °C and 109 °C, respectively, due to a cold crystallization phenomenon. Finally, two endothermic peaks, each representing their melting temperature, were detected around 148 °C and 154.5 °C for PLA and P-PLA, respectively. The double melting peak can be explained given that, at low scanning speed, there is sufficient time for thinner and less regular crystals to melt and then recrystallize. However, this phenomenon has also been attributed to other factors, as follows: (a) a low crystallization temperature, causing the presence of the alpha phase of the PLA; (b) the presence of more than one crystal structure; and (c) the presence of different lamellar morphologies formed before the heating phase [55]. The PLA peak at 333.79 °C can be ascribed to polymer degradation, according to [56]. The crystallinity of X_c_ of PLA and P-PLA is similar, around 12%.

Chitosan thermogram shows both endothermic and exothermic peaks. The endothermic peak at 100 °C is probably due to its melting transition [57]. The exothermic peak with an onset temperature (T^0^_d-Chi_) around 278 °C and a maximum at 306 °C indicates degradation due to dehydration and depolymerization [58] or the decomposition of amine units [59].

The hybrid scaffolds showed thermograms that can be described as the overlapping of the thermograms of the two components of PLA (or P-PLA) and Chi. As the melting peak of Chi is broad and close to the cold crystallization temperature of PLA and P-PLA, its presence partially hinders the cold crystallization peak of PLA and P-PLA. This effect is more evident when increasing the Chi concentration in the hybrid scaffolds. Similarly, in the region between 280 °C and 370 °C, the exothermic degradation peak of Chi is hindered by the more intense degradation endothermic peak of PLA, resulting in a pair of successive peaks, the first being exothermic (Chi degradation) and the second endothermic (PLA degradation). Interestingly, from Table 4 it is possible to observe that the onset of the Chi degradation peak shifted to lower temperatures when coated with PLA fibers. Furthermore, the T^0^_d-Chi_ decreased with increasing Chi concentration, suggesting that the Chi coating has a lower thermal stability than pristine Chi powder. This result can be ascribed to the partial degradation of the Chi molecules during the solvation process and to the increased specific surface of the material.

According to Equation (5), the crystallinity of PLA seems to increase with an increasing Chi concentration. However, this result can be ascribed mainly to the partial overlapping of the melting peak of Chi and the cold crystallization peak of PLA that resulted in an apparent decrease of the ΔH_cc_ of PLA and P-PLA. 

### 2.4. Chitosan Coating Resistance in PBS

The stability of PLA/Chi and P-PLA/Chi in PBS at 37 °C was evaluated according to Equation (4), assuming that all of the weight loss was ascribable to the chitosan release in the medium. This hypothesis was considered consistent as PLA and P-PLA scaffolds, used as controls, did not show any mass loss during the time points evaluated in this work (up to 144 h).

**Figure 7:**
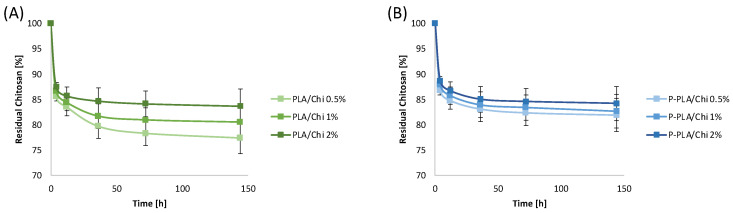
Percentage of residual chitosan weight on (**A**) PLA-based and (**B**) P-PLA-based scaffolds as a function of chitosan coating solution concentration and immersion time in PBS at 37°.

Results show that both PLA/Chi (Figure 7A) and P-PLA/Chi (Figure 7B) released about 13% of chitosan within the first 4 h. This burst of weight loss can be ascribed to the dissolution of low molecular weight chitosan molecules into the medium and was found to be independent from the substrate type and the chitosan concentration of the coating solution. Neither PLA/Chi 2% nor any of the P-PLA/Chi samples showed any significant change of residual chitosan percentage when increasing the testing time. On the other hand, PLA/Chi 0.5% and 1% showed further loss of chitosan up to 36 h of immersion, reaching a final value of residual chitosan equal to 77.4% and 80.5% after 144 h, respectively.

The plateau observed in the residual chitosan percentage curve displayed by all of the investigated scaffolds demonstrated the stability of the coating after 144 h of immersion. These results indicate that resistance of the chitosan coating in PBS at 37 °C was relatively high, although dependent on the substrate type and the initial amount of chitosan. In fact, the release rate of chitosan was slower for the P-PLA/Chi 0.5% and 1% systems than for the PLA/Chi 0.5% and 1% hybrid scaffolds. This result corroborates the hypothesis of higher affinity between chitosan and P-PLA when compared with the PLA substrate. In the PLA/Chi hybrid scaffold, the lower the chitosan concentration of the coating solution, the higher the release rate of chitosan. 

This result can likely be explained by considering that the lower the concentration of chitosan, the lower the fiber diameter of the hybrid scaffolds and, as a consequence, the higher the specific surface exposed to the medium. Furthermore, the residual chitosan values in PLA/Chi scaffold after immersion of 144 h were higher when increasing the Chi concentration in the coating solution. On the other hand, the P-PLA/Chi scaffold showed percentages of residual chitosan almost independently from the chitosan concentration used and higher than that of PLA/Chi samples.

### 2.5. Scaffolds Mineralization in SBF

**Figure 8:**
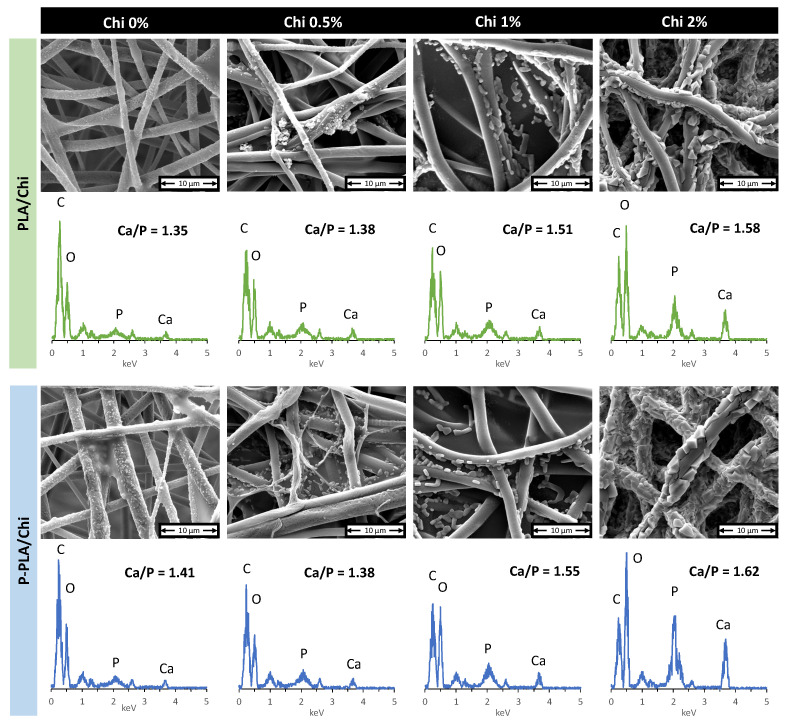
SEM images and EDS spectra of mineralized PLA and P-PLA fiber mat with and without Chi coating.

Figure 8 shows the SEM images and EDS spectra of mineralized PLA and P-PLA scaffolds with and without Chi coating. The images reveal the presence of highly roughened particles, ascribable to a mineral phase in all of the samples’ surfaces after immersion in SBF for 7 days. PLA and P-PLA scaffolds showed the formation of a high number of small (around 100–200 nm) and roundly shaped aggregates. The number and size of the aggregates increased with increasing Chi coating thickness, and their shape, size, and number are comparable between the PLA/Chi and P-PLA/Chi groups. Notably, the fiber surface is completely covered by a rough and thick layer of calcium phosphate aggregates in the PLA/Chi 2% and P-PLA/Chi 2% samples, resulting in a drastically changed morphology after immersion in SBF. 

The nature of the mineral phase was first studied by EDS, the spectra of which show that the major elements of the mineralized scaffold consisted of carbon (C), oxygen (O), phosphorus (P), and calcium (Ca), as shown in Figure 8. According to the materials used for the scaffolds’ fabrication, phosphorus and calcium could only derive from the mineral phase. This observation suggests that the mineral deposited on the surface of PLA fibers is a form of calcium phosphate. However, additional analysis is necessary to detail the characteristic of this mineral phase. EDS results were used to analyze the ratio of calcium and phosphate from the surface of the prepared materials. The Ca/P ratio increased from 1.35 to over 1.60 when the PLA and P-PLA scaffolds were coated with Chi. Moreover, samples with the thickest Chi coating exhibited a higher Ca/P ratio of around 1.6. The increase in the calcium phosphate deposition on chitosan-coated PLA scaffolds has already been observed in the literature [60], where it was ascribed to the increase in the hydrophilic properties of the Chi-coated PLA fibers.

### 2.6. Scaffolds Biocompatibility

The biocompatibility of the different types of PLA scaffolds was investigated both by indirect and direct tests on mouse pre-osteoblastic MC3T3-E1 cell line. The indirect test was performed by incubating P-PLA scaffolds in the culture medium for 72 h, which were then used for cell growth for another 24 h. Scaffolds alone or with chitosan added (0.5%, 1% and 2%) showed very good biocompatibility (viabilities of 108%, 98.5%, 97.5% and 100.7%, respectively) (Supplementary Materials, Figure S1).

**Figure 9:**
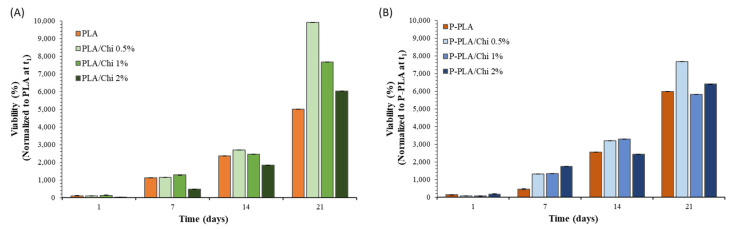
Alamar-Blue colorimetric assay of MC3T3-E1 cells grown on PLA, PLA/Chi 0.5%, PLA/Chi 1%, PLA/Chi 2% (**A**) and P-PLA, P-PLA/Chi 0.5%, P-PLA/Chi 1%, P-PLA/Chi 2% (**B**) for 1, 7, 14 and 21 days. Cell viability was expressed as percentage values normalized to PLA (**A**) or P-PLA (**B**) scaffolds at t_1_ (controls).

A biocompatibility direct assay was performed by seeding mouse pre-osteoblastic MC3T3-E1 cells and analyzing their viability and proliferation rate after different times (1, 7, 14 and 21 days). As reported in Figure 9, both PLA/Chi and P-PLA/Chi hybrid scaffolds present good biocompatibility and the capability to behave as substrates for cells. After 7 and 14 days, there was no significant difference in proliferation rate on PLA/Chi scaffolds, which became more evident, in a chitosan-dependent way, after 21 days (Figure 9A). According to the literature that has reported the chitosan effect on attachment and proliferation of many types of cells, including osteoblast [61,62,63], the addition of chitosan increases the cell viability compared with net PLA, and the PLA/Chi 0.5% sample presents the highest value. This result was also obtained for samples treated with plasma (Figure 9B), enhancing the biological compatibility and as indicated by the higher viability values of the P-PLA samples at 21 days compared with PLA at the same time (10,715.5 RFU; 5991.89% vs. 8931.4 RFU; 4994.26%, respectively). These data were also confirmed by fluorescence microscopy, where an increase in cell density over time was evident (Supplementary Materials, Figures S2–S9). These data can be related to several scaffold-related parameters, including the fiber diameter, the porosity, and the mechanical properties of the scaffolds [64]. Furthermore, the higher the chitosan concentration used in the coating solution, the higher the residual acetic acid (AA) traces in the chitosan coating. Although scaffolds were thoroughly washed in PBS before cell seeding, those AA traces can affect the cell viability of the MC3T3-E1 cells and explain the higher performance of PLA/Chi 0.5% and P-PLA/Chi 0.5% samples when compared with the others. 

**Figure 10:**
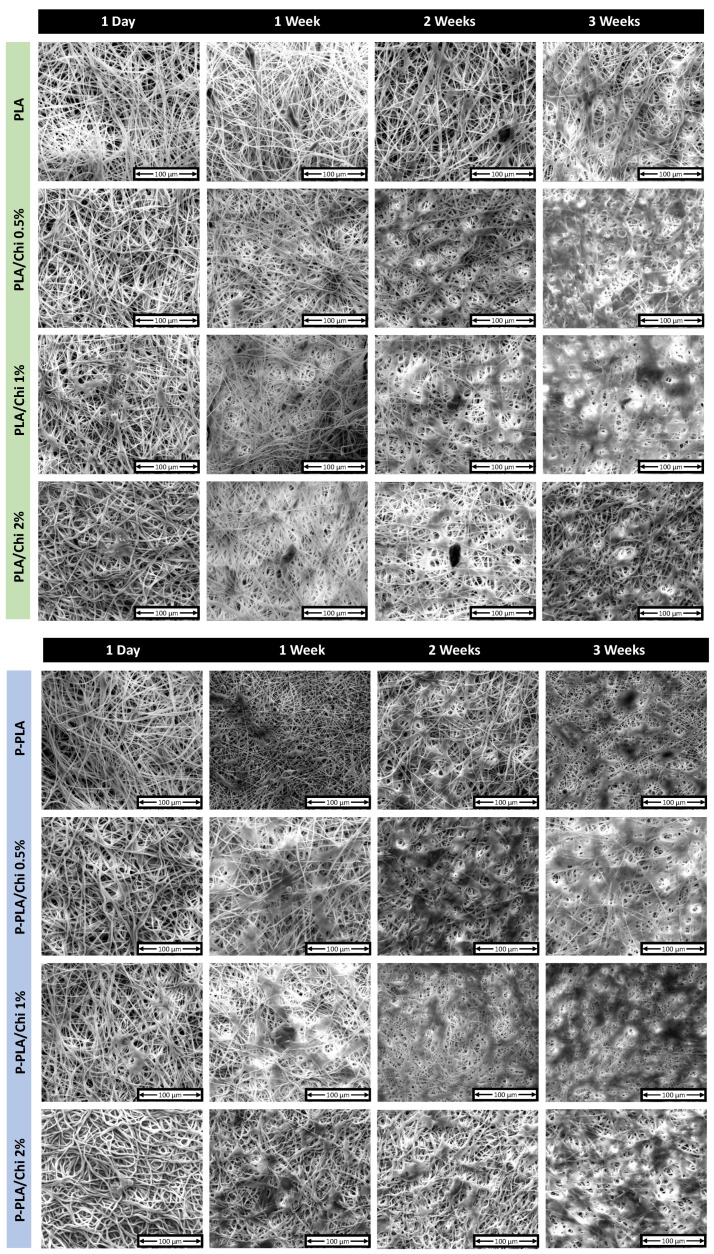
SEM micrographs of MC3T3 E1 cells grown on PLA, PLA/Chi 0.5%, PLA/Chi 1%, PLA/Chi 2%, P-PLA, P-PLA/Chi 0.5%, P-PLA/Chi 1%, and P-PLA/Chi 2% scaffolds for 1 (1 day), 7 (1 week), 14 (2 weeks) and 21 (3 weeks) days.

SEM images of cells grown on PLA/Chi and P-PLA/Chi hybrid scaffolds for up to 3 weeks are shown in Figure 10. 

The images corroborate the results of proliferation tests. In fact, the cell coverage on the scaffolds progressively increased as a function of time in all of the investigated samples. Furthermore, the cell shape suggests the fast attachment and the morphological adaptation of the cells on PLA, P-PLA and chitosan-coated fibers. After 1 day of seeding, single cells appeared attached to scaffold fibers; some cells still presented round morphology, while others started to spread between the fibers. At longer times (1 and 2 weeks) cells increased in number, starting to contact each other and forming a dense cell layer until the entire scaffolds were completely overgrown with cells after 3 weeks. The fibrillar structures of the scaffold allowed the identification of cells, thus suggesting a complete colonization of the scaffold.

### 2.7. Effect of Chitosan Coating and Plasma Treatment in Cell Differentiation

**Figure 11:**
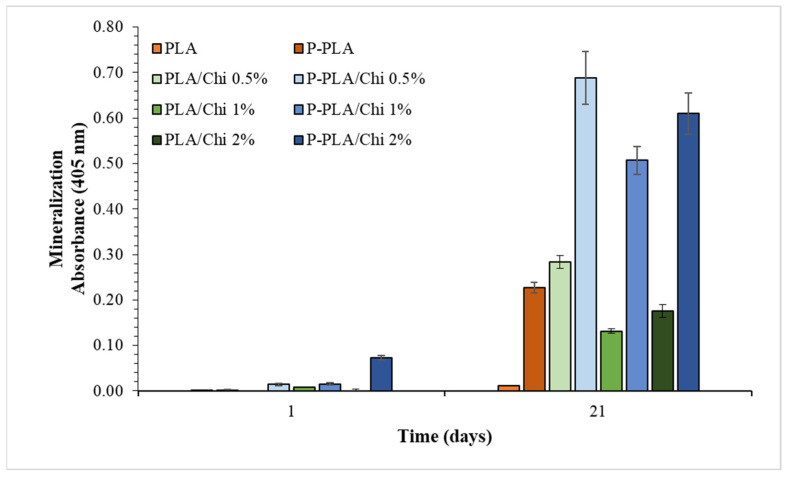
Alizarin Red S assay on MC3T3-E1 cells grown on PLA, PLA/Chi 0.5%, PLA/Chi 1%, PLA/Chi 2%, P-PLA, P-PLA/Chi 0.5%, P-PLA/Chi 1%, P-PLA/Chi 2% scaffolds for 1 day or 21 days.

One of the most important goals in bone tissue engineering is the possibility to induce osteoblast differentiation, the last step of which is matrix mineralization to maintain bone tissue mechanical integrity [65]. Alizarin Red S (ARS) assay, which permits the evaluation of the calcification, was performed on MC3T3-E1 cells grown on different scaffolds under calcification induction conditions. After 7 and 14 days, the values registered were the same as day 1, suggesting that the mineralization was not still occurring. After 21 days of seeding, an increase in calcium deposits was registered with some differences between the samples (Figure 11). Comparing PLA and PLA/Chi samples, the impact of chitosan on mineralization and osteoblast differentiation was evident; when PLA scaffolds were coated with CS (0.5%, 1% and 2%), the calcium deposits increased. Indeed, the osteogenic properties of chitosan are well known, so it is amply adopted in bone tissue engineering applications, even if CS alone is able to mimic all of the features of natural bone [66]. On the other hand, the mineralization was also increased by plasma treatment (0.01 vs. 0.226 for PLA and P-PLA samples, respectively), in accordance with the scientific literature [67,68,69,70]. This result can be ascribed to the changes in cell behavior due to differences on the initial protein adsorption through changes in surface energy and wettability observed in physiochemical characterization of the scaffolds [70]. 

Furthermore, plasma treatment probably influences and enhances the chitosan osteogenic properties of the scaffolds, as suggested by the high absorbance values registered (0.28 vs. 0.68, 0.13 vs. 0.50, and 0.18 vs. 0.60, for PLA/Chi 0.5% vs. P-PLA/Chi 0.5%, PLA/Chi 1% vs. P-PLA/Chi 1%, and PLA/Chi 2% vs. P-PLA/Chi 2%, respectively). 

**Figure 12:**
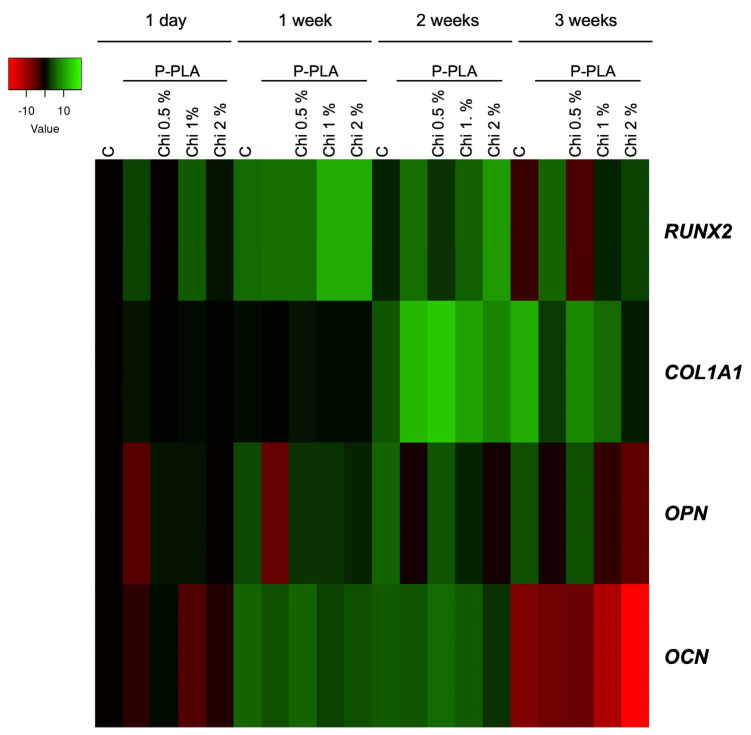
Heat map representation of the mean-centered data of RT-qPCR results showing the mRNA levels of analyzed genes in cell-grown P-PLA, with or without chitosan, at different percentages with respect to geometric averaging of 18S and GAPDH. Gene expression values are colored from red (low) to green (high). Fold changes in log (2)-transformed are represented. C: control, cells growth on 2D system. The unlabeled column refers to MC3T3 cells grown on P-PLA without chitosan. Gene expression results presented as heat maps were generated via Heatmapper available at http://heatmapper.ca/expression/ (accessed on 24 October 2023). The mRNA levels are represented as mean-centered, whereas the standard deviation (SD) (*n* = 3) was below 0.5%.

Starting from these results, a gene regulatory network involved in osteoblast differentiation was further investigated regarding P-PLA and P-PLA/Chi scaffolds (Figure 12). This includes runt-related transcription factor-2 (*RUNX2*), osteocalcin *(OCN*), osteopontin (*OPN*), and type 1 collagen (*COL1A1*) [71]. *RUNX2* is a transcription factor involved in the early stage of differentiation, as it is highly expressed in immature osteoblasts and downregulated in mature osteoblasts. *RUNX2* regulates the expression of other osteoblastic genes, including *COL1A1*, *OCN*, and *OPN*, which are involved in the intermediate and terminal stages of the osteogenic differentiation program, thus enabling extracellular matrix (ECM) deposition, bone mineralization and calcium ion homeostasis [72,73,74].

As reported in Figure 12, the mRNA levels of *RUNX2* were highest 1 week after induction, while an expected partial decrease was observed after 2 weeks in all analyzed samples. Moreover, and similar to the controls, *RUNX2* was downregulated in the osteoblasts on P-PLA/Chi 0.5% after 3 weeks, while it remained upregulated in the other samples. Because it has been reported that it acts as a master regulator of this program, the prolonged *RUNX2* overexpression in the other scaffolds suggests the occurrence of a certain difficulty in starting the osteoblast differentiation. 

Accordingly, as an early marker of osteoblast, *COL1A1* was upregulated in all of the experimental conditions, without significant changes among the used scaffolds. It has been shown that elevated *COL1A1* expression leads to a reduction in the early osteoblast markers, promoting enhanced maturation of bone cells. This suggests that cells can start the osteogenic commitment, enabling the progress towards the developmental program. Interestingly, the *OPN* mRNA levels increased and sustained until 3 weeks exclusively on cells growing on P-PLA/Chi 0.5%.

It is known that the expression of *OPN* increases concomitantly with the advancement in bone formation and mineralization [75]. *OPN*, a secreted phosphoprotein, takes a central role that remains only partially understood. Within the bone matrix, osteopontin engages with integrins via its arginine–glycine–aspartate (RGD) motif, facilitating the adhesion of bone cells to the mineral matrix, as reported by Morinobu et al. in 2003 [74]. Interestingly, studies on osteopontin-deficient mice have revealed an aberrant increase in mineral depositions compared with their wild-type counterparts [76]. Therefore, it is possible to hypothesize that the combination of plasma treatment and chitosan coating on PLA electrospun scaffolds positively addresses osteoblastic differentiation. 

## 3. Materials and Methods

### 3.1. Materials

PLA (2002D) was supplied by NatureWorks (Minnetonka, MN, USA) while high molecular weight chitosan, acetone (Ac), chloroforms (TCM), and glacial acetic acid (AA) were purchased from Sigma Aldrich (Saint Louis, MO, USA). 

### 3.2. PLA Electrospun Scaffolds Fabrication

A solution of PLA/TCM:Ac (2:1 vol) at 10 wt% of PLA was prepared at room temperature under continuous magnetic stirring overnight. The polymeric solution was placed into a 10 mL syringe fitted with a stainless-steel needle (19-G). The PLA scaffolds were prepared by setting the electrospinning apparatus (NF-103, MECC Co., Ltd., Fukuoka, Japan) according to the following parameters: solution flow rate = 0.7 mL/h; needle-collector distance = 15 cm; needle x-axes stage course = 8 cm; needle x-axes speed = 1 mm/min; high voltage = 15 kV, according to a previous work [8]. A grounded rotary drum (L = 20 cm, D = 10 cm, ω = 10 rpm) wrapped in an aluminum foil was used as a collector. The processing was carried out for 180 min to obtain PLA electrospun scaffolds about 50 µm thick. To remove any residual solvents, the collected samples were dried for 48 h under a fume hood.

### 3.3. Plasma Pretreatment of the PLA Scaffolds

A cold plasma reactor (Tucano, Gambetti, Italy) equipped with a polarized anode (RF = 13.56 MHz) set at 50 W for 30 s was used for the surface functionalization of the PLA electrospun scaffolds. The reaction was repeated twice to expose both surfaces of the PLA scaffolds to the electrode. Further processing carried out on plasma pretreated samples was executed within 30 min of the plasma treatment. The pretreated PLA scaffolds are coded as “P-PLA”.

### 3.4. Density, Porosity, and Water Uptake Measurements

The polymeric matrix density (*ρ_matrix_*) was measured as the average of ten measurements taken from each sample using a Pycnomatic ATC helium pycnometer (Thermo Electron Corporation, Waltham, MA, USA) whereas the apparent density of the scaffolds (*ρ_scaffold_*) was calculated by gravimetric analysis. The scaffold porosity was then evaluated according to the following Equation (1):

(1) $$Porosity\;\left(\%\right)=\left(1-\frac{\rho_{scaffold}}{\rho_{matrix}}\right)\times 100$$

The water uptake percentage of the PLA samples (*WU_PLA_*) was calculated via gravimetric measurements according to Equation (2):

(2) $${WU}_{PLA}\left(\%\right)=\frac{W_{wet}-W_{dry}}{W_{dry}}\times 100$$

where *W_wet_* and *W_dry_* are the weight of the PLA scaffolds after and before water absorption, respectively. A precision balance (Sartorius AX224, Goettingen, Germany, resolution of ±0.1 mg) was used for the gravimetric measurements.

### 3.5. Morphological Analysis

The morphology of the microfiber scaffolds was evaluated by scanning electron microscopy (SEM) (FEI Quanta 200 F, FEI, Hillsboro, OR, USA). The samples (3 mm diameter) were attached on an aluminum stub using an adhesive carbon tape. Samples were preliminarily gold sputter coated by means of a Sputtering Scancoat Six (Edwards Laboratories, Milpitas, CA, USA) for 60 s under argon atmosphere to avoid electrostatic discharge during the analysis. A plugin for ImageJ version 1.53a (Diameter J) [77] was used to determine the fiber diameter distribution and the mean fiber diameter of the scaffolds from the SEM micrographs via image processing (IP). A desktop scanning electron microscopy, (Phenom ProX, Phenom-World, Eindhoven, The Netherlands) equipped with an X-ray energy-dispersive probe (EDS) was used to evaluate the elemental composition of the samples surface. SEM and EDS analyses were performed on different areas of the samples in order to verify their homogeneity and uniformity.

### 3.6. Chitosan Solution Preparation and PLA Microfibers Coating

Electrospun PLA microfibers were coated with thin films of chitosan according to a preparation route described in a previous work [1]. In brief, according to assumptions that say that the whole PLA electrospun scaffold is composed of a single fiber (with a diameter equal to the mean diameter evaluated via IP) and that the density of PLA is known, it was possible to estimate the PLA fiber length. Three coating thicknesses were arbitrarily chosen, i.e., 85, 165, and 300 nm. Therefore, knowing the density of chitosan, the weight of chitosan needed to coat 1 g of electrospun scaffold was calculated for each desired thickness. As a consequence, three Chi/water:AA solutions were prepared at different Chi concentrations in a volume of solvent derived from the WU_PLA_. According to these theoretical evaluations, the weight percentage of Chi in the Chi/water:AA solutions were 0.5 wt%, 1.0 wt% and 2.0 wt% to theoretically reach the chitosan coating thicknesses of 85, 165, and 300 nm, respectively. The solubilization of chitosan was carried out in an aqueous AA solution (0.5 wt%) at 60 °C for 3 h under magnetic stirring according to [78,79]. A solution of 4 g of Chi/water:AA was placed in 0.1 g of electrospun PLA scaffolds until it was completely absorbed. Then, the wet scaffolds were left to dry under a fume hood overnight at room temperature, were washed in distilled water to remove any residual presence of AA and then dried again under a fume hood overnight at room temperature. The samples were coded as follows: PLA/Chi X% or P-PLA/Chi X% where X is 0.5, 1, or 2 on the basis of the Chi/water:AA solution used for the coating.

### 3.7. Chitosan Concentration in Hybrid Scaffolds

The chitosan weight concentration in PLA/Chi scaffolds was evaluated by gravimetric analysis according to Equation (3):

(3) $$Chi\;wt\%=\frac{W_{PLA/Chi}-W_{PLA}}{W_{PLA/Chi}}\times 100$$

where *W_PLA/Chi_* is the weight of the scaffold after chitosan coating and *W_PLA_* is the weight of the scaffold before coating.

### 3.8. Water Contact Angle Measurements

The wettability of the produced scaffolds was evaluated by the sessile drop method. Static water or SBF contact angle (WCA or SBFCA) measurements were carried out with an FTA 1000 (First Ten Ångstroms, Portsmouth, VA, USA) by using distilled water or SBF as fluid. The volume of the test liquid was 8 µL for all measurements and the value of the WCA was registered after the drop stabilized on the surface for 5 s according to [79]. Different drop images (7 spots for each sample) were taken.

### 3.9. Mechanical Properties

A universal testing machine (UTM, model 3367, Instron, Norwood, MA, USA), equipped with a 1 kN load cell and with a BioPulse bath, was used do carry out tensile mechanical measurements on rectangular-shaped specimens (10 × 90 mm, ~50 µm thick) that had been cut from the collected material along the radial direction of the rotary drum collector. The tests were performed at room temperature without the use of the bath (dry condition) and with samples immersed in PBS at 37 °C (wet condition). In the wet condition, the samples were conditioned for 30 min in PBS at 37 °C before the test. The measurements were performed according to the following parameters: crosshead speed 1 mm/min and gauge length 20 mm. The representative nominal stress–strain curves were reported for each scaffold. The elastic moduli of the samples were evaluated as the slopes of the linear regions of the stress–strain curves. Seven specimens were tested for each material and the average values of the mechanical parameters were reported with the relative standard deviations.

### 3.10. FT-IR/ATR Analysis

FT-IR/ATR analysis (FT-IR/NIR Spectrum 400 spectrophotometer from Perkin-Elmer Inc., Wellesley, MA, USA) was performed to investigate the sample chemical surface properties. For each sample, 4 accumulation scans with a resolution of 4 cm^−1^ were collected in the range 4000–500 cm^−1^.

### 3.11. X-ray Photoelectron Spectroscopy Analysis

X-ray photoelectron spectroscopy (XPS) was performed on PLA and P-PLA electrospun scaffolds. Spectra were recorded with a PHI 5000 VersaProbe II scanning XPS Microprobe (TM) using monochromatic Al-Kα radiation (h = 1486.6 eV) from an X-ray source operating at 200 μm spot size, 30 W power, and 15 kV acceleration voltage. The (iterative) Shirley background subtraction and the peak fitting with Gaussian–Lorentzian-shaped profiles were performed for the high-resolution XPS spectra analysis using Multipak software version 9.6.0.15 (ULVAC-PHI). The detected spectra were shifted to coincide with the C 1 s hydrocarbon peak at 285 eV. To resolve and analyze the chemical bonding states of the carbon atoms, a Shirley type background was subtracted and Gaussian–Lorentzian peak components were fitted to the C 1 s high-resolution spectra.

### 3.12. Chitosan Stability

To evaluate the stability of the chitosan coating on the PLA and P-PLA scaffolds, pre-weighted PLA/Chi and P-PLA/Chi scaffolds (*W*_1_) were immersed in PBS at 37 °C at different time points. Wet samples were extracted, gently washed in distilled water, and left to dry under a fume hood overnight. Finally, the dry samples where weighted (*W*_2_) and the residual chitosan % was obtained by assuming that all of the weight-loss was ascribable to the chitosan release in water according to Equation (4):

(4) $$Residual\;Chitosan\;[\%]=\left(\frac{{W_{Chi}+W}_{2}-W_{1}}{W_{Chi}}\right)\times 100$$

where *W_Chi_* is the weight of chitosan on the PLA (or P-PLA) scaffolds according to the Chi wt% evaluated via Equation (3). 

As control, PLA and P-PLA scaffolds that underwent the same treatments were used.

### 3.13. Differential Scanning Calorimetry

Differential scanning calorimeter (DSC), (Setaram, Caluire, France, model DSC131) was used to investigate the calorimetric properties of the scaffolds. The analysis was carried out with two cycles of heating from room temperature to 400 °C at 10 °C/min heating rate under nitrogen flow on electrospun samples with approximately the same weight (~5 mg) sealed in aluminum pans.

PLA crystallinity degree was calculated according to Equation (5) [80]:

(5) $$\chi_{c}\;\left(\%\right)=\frac{{∆H}_{m}-{∆H}_{cc}}{{{∆H}^{0}}_{PLA\;}\times X_{PLA}}\times 100$$

where Δ*H_cc_* and Δ*H_m_* are the cold crystallization and melting enthalpy of the samples, respectively, *X_PLA_* is the weight fraction of PLA, and ΔH^0^_m_ is the melting enthalpy of 100% crystalline PLA equal to 93.7 J/g [80].

### 3.14. Incubation of Scaffold in SBF

The scaffolds for the mineralization study were cut into a cylindrical shape with dimeter equal to 15 mm. Each sample was incubated in a 3 mL solution of SBF maintained at 37 °C for 7 days for the mineral growth. If necessary, a series of brief evacuation–repressurization cycles was performed to force the solution into the pores of the scaffold. The SBF solution was renewed every 2 days, according to [81]. After that, the samples were gently rinsed with distilled water and then dried overnight. The SBF solution was prepared according to the procedure reported in [82].

### 3.15. Biological Evaluation

For the biological evaluations, scaffolds (PLA, PLA/Chi 0.5%; PLA/Chi 1%; PLA/Chi 2%; P-PLA; P-PLA/Chi 0.5%; P-PLA/Chi 1%; P-PLA/Chi 2%) were sterilized by UV (275 nm) treatment for 2 h (1 h on each side) under a laminar fume hood and pre-conditioned overnight with complete culture medium to be completely imbibed. This step is essential to promote cell adhesion; if the scaffolds are not well soaked, a low seeding yield is obtained. Mouse pre-osteoblastic MC3T3-E1 cell line (ECACC, European Collection of Cells Cultures) was grown in Dulbecco’s modified Eagle’s high glucose medium (DMEM, Sigma Aldrich) supplemented with 10% (*v*/*v*) fetal bovine serum (FBS) (Euroclone, Celbar), 100 units per ml penicillin G, 100 μg/mL streptomycin (Euroclone, Celbar) and 2 mM L-glutamine (Euroclone, Celbar) at 37 °C, in a humidified atmosphere of 5% CO_2_.

An amount of 5 × 10^3^ cells was resuspended in 5 µL of complete medium, corresponding to the volume of the scaffold seeded on each sample and incubated in a humidified incubator (37 °C; 5% CO_2_). After the 30 min required for the cell adhesion, each scaffold was transferred to one well of a 48-well plate added with DMEM complete medium which was refreshed every three days.

### 3.16. Cell Proliferation Assay

At different times (1, 7, 14 and 21 days) from cell seeding, the viability and cell proliferation rate for each type of electrospinning device were evaluated using alamarBlue colorimetric assay (Thermo Scientific, Foster City, CA, USA) according to the manufacturer’s recommendations. Cells grown on each type of scaffold were incubated with Alamar-Blue reagent solution (10% in culture medium) in a complete medium for 2 h in a humidified incubator (37 °C; 5% CO_2_). 

Fluorescence intensity (λ_exc_ 530/25 nm and λ_emm_ 590/35 nm expressed as relative fluorescent units (RFU)), which changes according to the degree of cell viability, was evaluated through a microplate reader (Synergy HT, Biotek, Winooski, VT, USA). The fluorescence values of each type of scaffold were normalized against the same type of unseeded device, used as a blank. The assay was performed in quadruplicate for each scaffold formulation and repeated three times. The cell viability was expressed as a percentage normalized to PLA or P-PLA scaffolds at time 1 (t_1_). 

### 3.17. SEM Analysis on Cellularized Scaffolds

Scanning electron microscope (SEM) analysis was performed on each type of scaffold (PLA, PLA/Chi 0.5%; PLA/Chi 1%; PLA/Chi 2%; P-PLA; P-PLA/Chi 0.5%; P-PLA/Chi 1%; P-PLA/Chi 2%) in which cells were grown for 1, 7, 14 and 21 days. After appropriate washing in PBS, samples were fixed with 4% (*v*/*v*) glutaraldehyde at 4 °C for 2 h. Then, samples were rinsed with water and dehydrated with ethanol series (25%, 50%, 75% *v*/*v* and pure ethanol). Therefore, they were dried under prior vacuum gold-sputtering and analyzed by SEM.

### 3.18. Mineralization Detection and Quantification: Alizarin Red S Assay

After 1 day of seeding, mineralization was induced on MC3T3-E1 cells grown in a 2D system (on plate) or on scaffolds by adding DMEM containing 10% (*v*/*v*) fetal bovine serum (FBS) (Euroclone, Celbar), 100 units per ml penicillin G, 100 μg/mL streptomycin (Euroclone, Celbar) and 2 mM L-glutamine (Euroclone, Celbar) and the following osteogenic supplements: β-glycerophosphate (10 mM, Sigma Aldrich) and ascorbic acid (50 mg/mL, Sigma Aldrich). Cultures were incubated at 37 °C with 5% CO_2_, changing the medium every 3 days. After 1, 7, 14 and 21 days of seeding (T1; T7; T14; T21) the mineralization rate was quantified in each type of scaffold (PLA, PLA/Chi 0.5%; PLA/Chi 1%; PLA/Chi 2%; P-PLA; P-PLA/Chi 0.5%; P-PLA/Chi 1%; P-PLA/Chi 2%) by using the Alizarin Red S (ARS) stain that permits evaluation of any calcium deposits. Calcium forms an Alizarin Red S–calcium complex that presents intensive red staining. An amount of 1 mL of ARS (40 mM, pH 4.1) was added to each sample and incubated at room temperature for 20 min with gentle shaking. After the remotion of unincorporated dye, the samples were thoroughly washed with dH_2_O in agitation for 15 min. For the quantification of the mineralization rate, each type of scaffold was finely chopped with a scalpel and moved to a 1.5 mL microcentrifuge tube with 800 mL of 10% (*v*/*v*) acetic acid and incubated at room temperature for 30 min with soft shaking. After vortexing for 30 s, the slurry was overlaid with 500 mL of 1-Butanol (Sigma Aldrich), heated at 85 °C for 10 min and transferred to an ice bath until completely cooled (5 min). The slurry was then centrifuged at 14,000 RPM for 15 min to obtain a phase inversion; 500 mL of the supernatant was transferred to a new 1.5 mL microcentrifuge tube and 100 mL of NaOH 1N was added to neutralize the acid. The pH was measured to ensure that it was between 4.1 and 4.5. Aliquots (150 mL) of the supernatant were read in triplicate in a 96-well plate by a microplate reader (Synergy HT, Biotek, Winooski, VT, USA) at the wavelength of 405 nm. 

### 3.19. RNA Isolation and cDNA Synthesis

Total RNA was purified using Trizol (Invitrogen, Waltham, MA, USA), according to the manufacturer’s instructions, from MC3T3-E1 cells cultured on different supports including Petri dishes, and all the different types of PLA scaffolds above defined at days 1, 7, 14, and 21. RNA concentrations and quality were verified by measuring the optical density at Abs260 nm and Abs260/280 nm. The RNA integrity was evaluated by denaturing 1.5% agarose gel. To remove any residual trace of DNA contamination, 500 ng of extracted RNA was digested with DNase RQ1 RNase-Free (Promega, Madison, WI, USA) for 30 min at 37 °C, while DNase was inactivated by adding 25 mM EDTA. First-strand cDNA was synthesized from 250 ng DNase-treated RNA as a template in the presence of random primers and using the SuperScript III First-Strand Synthesis System (Life Technologies Corporation, Carlsbad, CA, USA) according to the manufacturer’s instructions. The cDNA mixtures were then stored at −20 °C until used.

### 3.20. qPCR Analyses

The qPCR analyses were carried out on a BIO-RAD CFX96 System using BlasTaq™ 2X qPCR MasterMix (Applied Biological Materials Inc., Richmond, BC, Canada) as detection chemistry. Real-time PCRs were performed in a 15 µL mixture containing 2 µL of a 1:5 dilution of the cDNA preparations. The following parameters were used for qPCR: 95 °C for 3 min, followed by 40 cycles of 95 °C for 15 s and 60 °C for 60 s, followed by melting curve analysis and electrophoresis on 2% agarose gels to confirm the absence of nonspecific products. Primer pairs used in this study are shown in Table 5. The *18S rRNA*, and *GAPDH* were chosen as reference genes. A normalization factor was calculated based on geometric averaging of the expression level of these reference genes and was used to quantify the expression levels of the target genes using the −ΔΔCt method. Amplifications were run in triplicate. All data represent relative mRNA expressed as the mean ± S.D. (*n* = 3). Significant differences among groups were determined using one-way ANOVA using Statistica 6.0 (StatSoft, Tulsa, OK, USA).

### 3.21. Statistical Analysis

Statistical analyses of the data were performed through one-way analysis of variance, and when applicable, data were compared using the Student’s *t*-test. *p*-value < 0.05 was considered statistically significant. ANOVA was employed to evaluate any significant differences between the values obtained during characterization of the samples. A *p* value < 0.05 was considered significant.

## 4. Conclusions

In this work, core–shell microfibrous scaffolds composed of PLA and chitosan were fabricated via direct coating for bone tissue engineering applications and characterized for their physical and biological properties. The effect of an air–plasma treatment on the PLA fibers and of the concentration of chitosan in the coating solution was investigated and evaluated in terms of the final scaffolds’ properties.

The effective chitosan coating was confirmed by FTIR-ATR, morphological, EDS, DSC, and gravimetric analyses. These results suggest the fine control of the composition of the final hybrid scaffolds by simply modifying the chitosan concentration in the coating solution. SEM investigation and image processing analysis confirmed that chitosan coating wrapped the electrospun fibers, resulting in a core–shell structure that increased their wettability. The mechanical analyses revealed that Chi coating increased the elastic modulus of the scaffolds. These results are more pronounced at higher chitosan concentrations and when the PLA fibers have been pretreated via air–plasma, probably due to a higher affinity between the phases that is itself due to the oxygenated moieties on the PLA surfaces. Plasma pretreatment was also able to increase the coating resistance in PBS at 37 °C, although all of the samples showed high coating stability up to 144 h. All Chi-coated scaffolds displayed increased calcium phosphate aggregates with increasing Ca/P ratio after 7 days of incubation in SBF.

Biological evaluation performed by seeding mouse pre-osteoblastic MC3T3-E1 cells highlighted the good biocompatibility and the chitosan effect in cell proliferation. Furthermore, SEM and fluorescence analyses showed cell colonization and distribution over time. On the other hand, cell differentiation was investigated both by biochemical (Alizarin Red assay) and molecular (real-time PCR) analyses. Data suggest that the combination of plasma treatment and chitosan functionalization of PLA scaffolds positively influences osteoblastic differentiation via calcium deposits and activation of a specific regulatory network. Therefore, evidence that P-PLA/chitosan hybrid scaffolds are suitable candidates for bone tissue engineering emerged.

## Figures and Tables

**Table 1:** Surface elemental composition from wide-scan XPS and fraction of carbon functional groups from high resolution C 1s peaks.

Sample	C 1s (%)	O 1s (%)	O/C	C–C (%) ∼285 eV	C–O (%) ∼285 eV	O–C=O (%) ∼288.9 eV
PLA	64.77	35.01	0.54	49.23	25.98	24.81
P-PLA	62.02	37.21	0.60	40.43	29.74	30.12

**Table 2:** EDS results for PLA/Chi hybrid scaffolds at different chitosan concentrations with and without plasma pretreatment.

Sample	C (wt%)	O (wt%)	N (wt%)
PLA	17.1	82.9	0
PLA/Chi 0.5%	15.1	81.3	3.6
PLA/Chi 1%	15	81.3	3.7
PLA/Chi 2%	14.9	81.3	3.8
P-PLA	16.7	83.3	0
P-PLA/Chi 0.5%	15.2	81.2	3.6
P-PLA/Chi 1%	14.9	81.3	3.8
P-PLA/Chi 2%	14.9	81.2	3.9

**Table 3:** Tensile properties of the PLA/Chi and P-PLA/Chi hybrid scaffolds in dry and wet testing conditions.

	E (MPa)	TS (MPa)	ε_b_ (%)
Dry condition			
PLA	20.18 ± 1.21 ^a^	1.64 ± 0.19 ^a^	148.61 ± 11.40 ^a^
PLA/Chi 0.5%	37.45 ± 2.45 ^b^	2.65 ± 0.31 ^b^	22.11 ± 1.74 ^b^
PLA/Chi 1%	87.54 ± 4.76 ^c^	2.97 ± 0.30 ^b^	19.31 ± 1.53 ^b^
PLA/Chi 2%	259.02 ± 15.13 ^d^	2.85 ± 0.18 ^b^	2.57 ± 0.28 ^c^
P-PLA	20.95 ± 1.54 ^a^	1.16 ± 0.17 ^e^	153.70 ± 11.06 ^a^
P-PLA/Chi 0.5%	41.11 ± 3.04 ^e^	2.86 ± 0.18 ^b^	14.77 ± 1.13 ^d^
P-PLA/Chi 1%	110.57 ± 7.58 ^f^	3.75 ± 0.29 ^c^	11.09 ± 1.07 ^e^
P-PLA/Chi 2%	892.64 ± 58.49 ^g^	7.37 ± 0.49 ^d^	1.76 ± 0.11 ^f^
In PBS at 37 °C			
PLA	17.01 ± 1.01 ^a^	1.02 ± 0.13 ^a^	238.61 ± 23.42 ^a^
PLA/Chi 0.5%	22.97 ± 2.78 ^b^	1.21 ± 0.18 ^a^	132.11 ± 12.04 ^b^
PLA/Chi 1%	28.23 ± 3.96 ^c^	2.27 ± 0.20 ^b^	113.31 ± 9.98 ^b,c^
PLA/Chi 2%	52.32 ± 5.66 ^d^	2.45 ± 0.22 ^b^	90.18 ± 6.28 ^d^
P-PLA	18.15 ± 1.14 ^a^	0.91 ± 0.11 ^a^	249.37 ± 21.06 ^a^
P-PLA/Chi 0.5%	20.50 ± 2.12 ^b^	1.51 ± 0.19 ^c^	120.14 ± 11.30 ^b^
P-PLA/Chi 1%	34.39 ± 3.98 ^e^	2.01 ± 0.21 ^b^	110.9 ± 9.07 ^c^
P-PLA/Chi 2%	107.57 ± 13.51 ^f^	2.09 ± 0.24 ^b^	93.09 ± 8.83 ^d^

Each value is the mean of 5 replicated samples. For each testing condition, values in a column followed by different letters are significantly different at *p* < 0.05.

**Table 4:** Thermal properties of the PLA and P-PLA in PLA/Chi and P-PLA/Chi scaffolds.

	T_g_ (°C)	T_cc_ (°C)	T_m1_ (°C)	T_m2_ (°C)	T^0^_d-Chi_ (°C)	ΔH_cc_ (j/g)	ΔH_m_ (j/g)	X_c_ (%)
PLA	64.70	102.22	148.06	154.57	-	19.12	30.70	12.36
PLA/Chi 0.5%	64.48	111.67	148.11	155.31	273	12.11	21.23	13.79
PLA/Chi 1%	61.66	106.17	148.03	154.58	269	5.72	17.48	23.06
PLA/Chi 2%	60.17	-	-	154.57	258	-	11.68	32.65
P-PLA	64.23	108.84	147.98	154.38	-	19.31	31.00	12.48
P-PLA/Chi 0.5%	63.40	111.68	148.22	155.09	272	13.01	21.11	12.25
P-PLA/Chi 1%	62.59	101.18	147.64	155.08	268	9.20	17.3	15.88
P-PLA/Chi 2%	62.01	-	150.54	155.07	265	3.22	12.21	25.13

**Table 5:** Oligonucleotides set used in this study.

Gene Name	Primers Sequence	Gene Bank Accession Number
*18S rRNA*	GCAATTATTCCCCATGAACG ^a^ GGCCTCACTAAACCATCCAA ^b^	NR_003278.3
*GAPDH*	CATCACTGCCACCCAGAAGACTG ^a^ ATGCCAGTGAGCTTCCCGTTCAG ^b^	NM_001289726.2
*OPN*	GCTTGGCTTATGGACTGAGGTC ^a^ CCTTAGACTCACCGCTCTTCATG ^b^	NM_001204201.1
*COL1A1*	TTCTGTGGGTCCTGCTGGGAAA ^a^ TTGTCACCTCGGATGCCTTGAG ^b^	NM_007742.4
*RUNX2*	CCTGAACTCTGCACCAAGTCCT ^a^ TCATCTGGCTCAGATAGGAGGG ^b^	NM_001145920.2

^a^ Forward primer, ^b^ reverse primer.

## Data Availability

The data presented in this study are available on request from the corresponding author.

## Supplementary Materials

The following supporting information can be downloaded at: https://www.mdpi.com/article/10.3390/ijms25052507/s1.
