# Supplementary material for: Improvement of Osteogenic Differentiation of Mouse Pre-Osteoblastic MC3T3-E1 Cells on Core–Shell Polylactic Acid/Chitosan Electrospun Scaffolds for Bone Defect Repair
Source: Int J Mol Sci. 2024 Feb 21;25(5):2507. doi: 10.3390/ijms25052507 (PMC10931705; doi:10.3390/ijms25052507)
Supplement: Supplementary file 1 [file ijms-25-02507-s001.zip › ijms-2883620-supplementary.pdf]

Supporting information

Improvement of osteogenic differentiation of mouse pre-osteoblastic MC3T3-E1 cells on core-shell Polylactic acid/Chitosan electrospun scaffolds for bone defect repair

Francesco Lopresti[1,*,†], Simona Campora[2,*,†], Salvatrice Rigogliuso[2], Aldo Nicosia[2,3], Alessandra Lo Cicero[2], Chiara Di Marco[1], Salvatore Tornabene[1], Giulio Ghersi[2,4], Vincenzo La Carrubba[1,5]

[1]Department of Engineering, University of Palermo, RU INSTM, Viale delle Scienze, Palermo, Italy

[2]Department of Biological, Chemical and Pharmaceutical Sciences and Technologies [STEBICEF] University of Palermo, Viale delle Scienze, Ed. 16, 90128 Palermo, Italy.

[3] Institute for Biomedical Research and Innovation, Italian National Research Council (IRIB-CNR), 90146 Palermo, Italy

[4]Abiel s.r.l, c/o University of Palermo, Viale delle Scienze, Ed. 16, 90128 Palermo, Italy;

[5]ATeN Center, University of Palermo, Viale delle Scienze, Palermo, Italy

* Corresponding authors: Francesco Lopresti, email: francesco.lopresti01@unipa.it; Simona Campora, email: simona.campora@unipa.it

[†] These authors contributed equally to this work

Materials and methods

Scaffold Biocompatibility by cell viability assay

Following the sterilisation process by UV treatment (254 nm) for 2 hours (1 hour on each side) under a laminar fume hood), scaffold biocompatibility was performed by the indirect method by incubating P-PLA; P-PLA/Chi 0.5%; P-PLA/Chi 1% or P-PLA/Chi 2% scaffolds in Dulbecco's modified Eagle's-high glucose medium (DMEM, Sigma Aldrich) supplemented with 10% (v/v) fetal bovine serum (FBS) (Euroclone, Celbar), 100 units per ml penicillin G, 100 µg/ml streptomycin (Euroclone, Celbar) and 2 mM L-glutamine (Euroclone, Celbar) for 72 hours (named scaffold medium). 10×10^3 MC3T3-E1 cells were seeded into 96 well plates and growth in Dulbecco's modified Eagle's-high glucose medium (DMEM, Sigma Aldrich) supplemented with 10% (v/v) fetal bovine serum (FBS) (Euroclone, Celbar), 100 units per ml penicillin G, 100 µg/ml streptomycin (Euroclone, Celbar) and 2 mM L-glutamine (Euroclone, Celbar) at 37°C in a

humidified atmosphere of 5% CO_2 for 24 hours and then incubated with scaffold medium and growth for 24hours. Therefore, Alamar blue colorimetric assay (Thermo Scientific, Foster City, CA, USA) was performed by incubating cells with Alamar-Blue reagent solution (10% in culture medium) for 2 h in a humidified incubator (37°C; 5% CO_2). Fluorescence intensity (λ_{exc} 530/25 nm and λ_{emm} 590/35 nm), which changes according to the degree of cell viability, was evaluated through a microplate reader (Synergy HT, Biotek, Winooski, VT, USA) and expressed as a viability percentage established cells growth with DMEM medium as control (100%).

Scaffold Biocompatibility by fluorescence microscopy

After 1, 7, 14 and 21 days of seeding, MC3T3-E1 cells grown in 3D conditions on each type of electrospinning device (PLA, PLA/Chi 0.5%; PLA/Chi 1%; PLA/Chi 2%; P-PLA; P-PLA/Chi 0.5%; P-PLA/Chi 1%; P-PLA/Chi 2%), were fixed with 3.7% formaldehyde for 10 minutes at room temperature. Subsequently, the samples were washed thoroughly with PBS (Phosphate Buffer Saline) and stained with DAPI (1:10.000 Sigma, Milan, Italy; blue nuclei) for 15 min at 37°C. The samples were analyzed by fluorescence microscopy (Leica). PLA, PLA/Chi 0.5%; PLA/Chi 1%; PLA/Chi 2%; P-PLA; P-PLA/Chi 0.5%; P-PLA/Chi 1%; P-PLA/Chi 2% scaffolds without cells were used as a control to set the laser intensity.

Results

Scaffold Biocompatibility

Scaffold biocompatibility was performed by indirect method by cell viability assay (Figure S1). After incubation with all the scaffold mediums (P-PLA; P-PLA/Chi 0.5%; P-PLA/Chi 1%; P-PLA/Chi 2%), cells showed good viability (viability of 108%, 98.5%, 97.5% and 100.7%, respectively), suggesting an excellent biocompatibility.

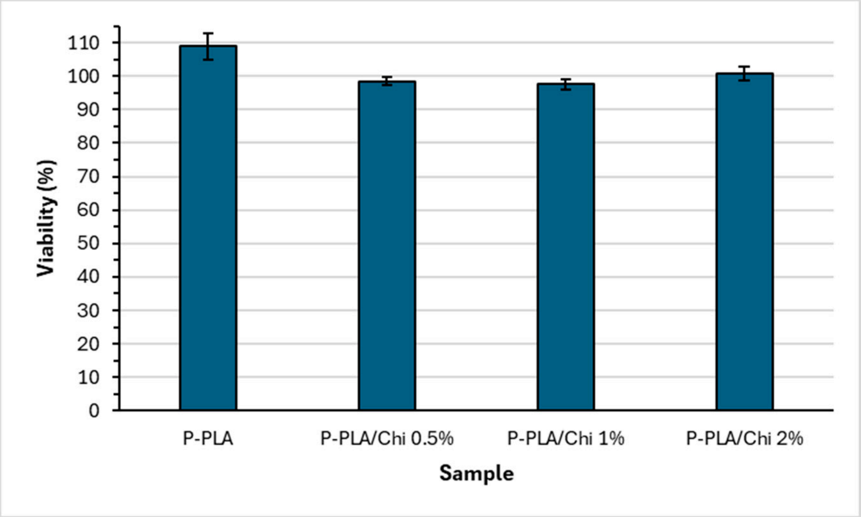

Figure S1. Alamar-Blue assay of MC3T3-E1 cells grown on P-PLA, P-PLA/Chi 0.5%, P-PLA/Chi 1%, P-PLA/Chi 2% medium for 24 hours. Cell viability was expressed as a percentage indicating the cells grown in DMEM as control (100%).

Furthermore, scaffolds biocompatibility was further investigated by fluorescence analysis of MC3T3-E1 cells seeded on different types of scaffolds over time (1, 7, 14 and 21 days). After one day of seeding, cells grown on PLA electrospinning devices coated with different concentrations of chitosan (0,5%, 1% and 2%, respectively PLA/Chi 0.5%; PLA/Chi 1% and PLA/Chi 2%) were attached to the samples (Figure S2, S3, S4 and S5). The cell densities increased in a time-dependent way, suggesting that cells can proliferate on the scaffolds and colonize them.

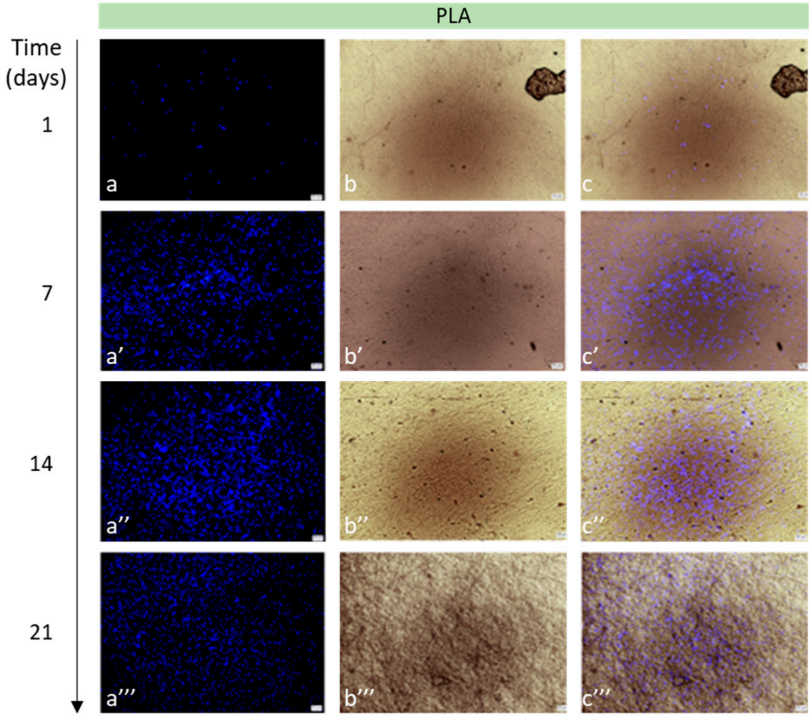

Figure S2. Fluorescence microscopy of MC3T3-E1 cells grown on PLA scaffolds for 1, 7, 14 and 21 days. Blue: nuclei, DAPI (a, a', a'', a'''), light (b, b', b'', b''') and merge (c, c', c'', c'''). Magnification 10X.

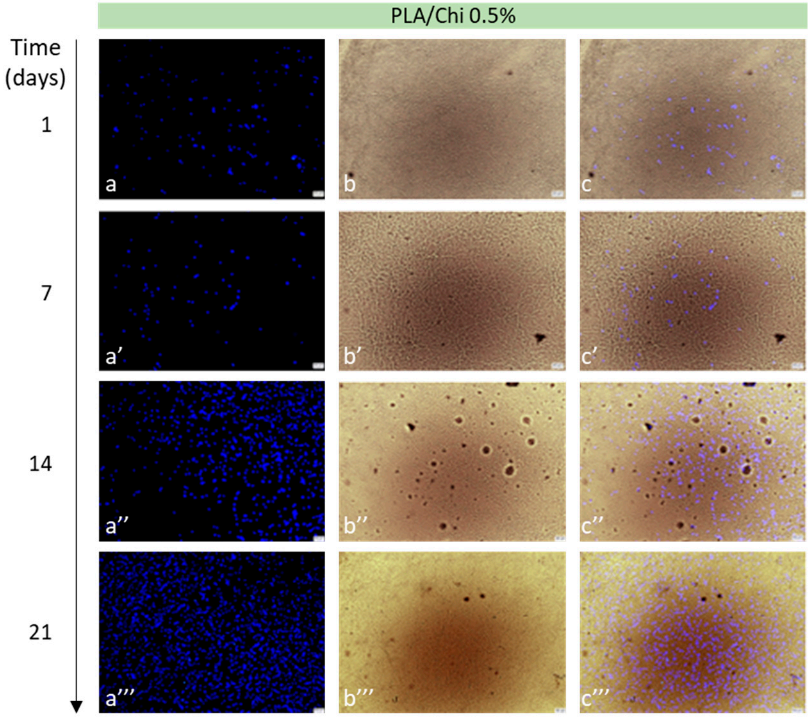

Figure S3. Fluorescence microscopy of MC3T3-E1 cells grown on PLA/Chi 0.5% scaffolds for 1, 7, 14 and 21 days. Blue: nuclei, DAPI (a, a', a'', a'''), light (b, b', b'', b''') and merge (c, c', c'', c'''). Magnification 10X.

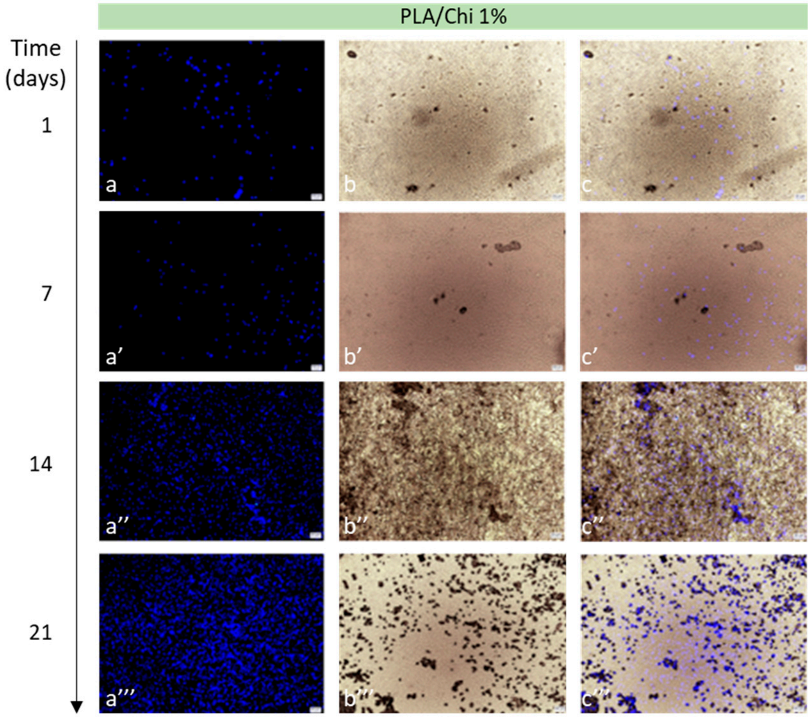

Figure S4. Fluorescence microscopy of MC3T3-E1 cells grown on PLA/Chi 1% scaffolds for 1, 7, 14 and 21 days. Blue: nuclei, DAPI (a, a', a'', a'''), light (b, b', b'', b''') and merge (c, c', c'', c'''). Magnification 10X.

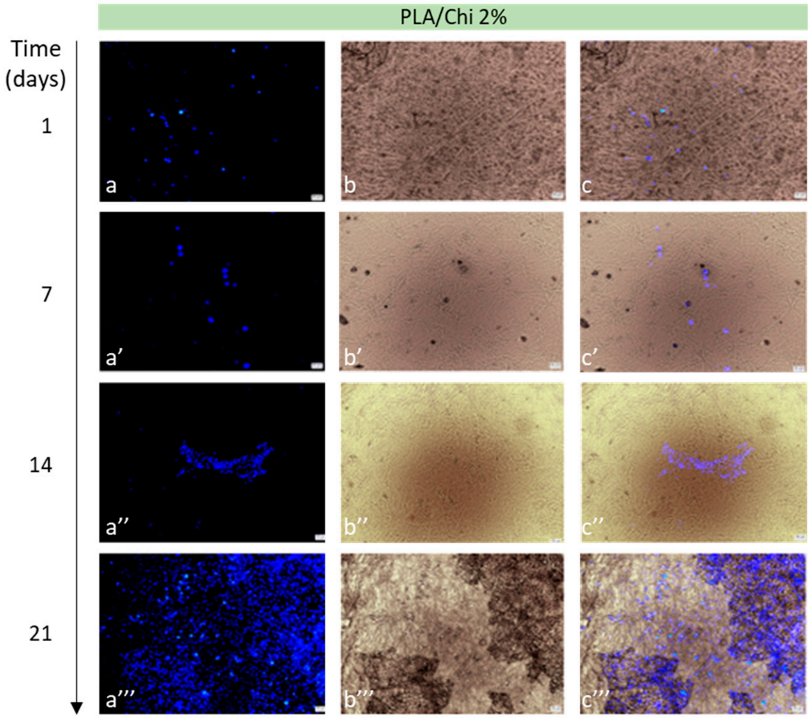

Figure S5. Fluorescence microscopy of MC3T3-E1 cells grown on PLA/Chi 2% scaffolds for 1, 7, 14 and 21 days. Blue: nuclei, DAPI (a, a', a'', a'''), light (b, b', b'', b''') and merge (c, c', c'', c'''). Magnification 10X.

The same trend was obtained following the cell colonization on P-PLA scaffolds coated with chitosan (0,5%, 1% and 2%, respectively P-PLA/Chi 0.5%; P-PLA/Chi 1% and P-PLA/Chi 2%) in which it was evident the increase of the blue fluorescence relative to cell nuclei over time (1, 7, 14 and 21 days) suggesting cell proliferation (Figure S6, S7, S8 and S9).

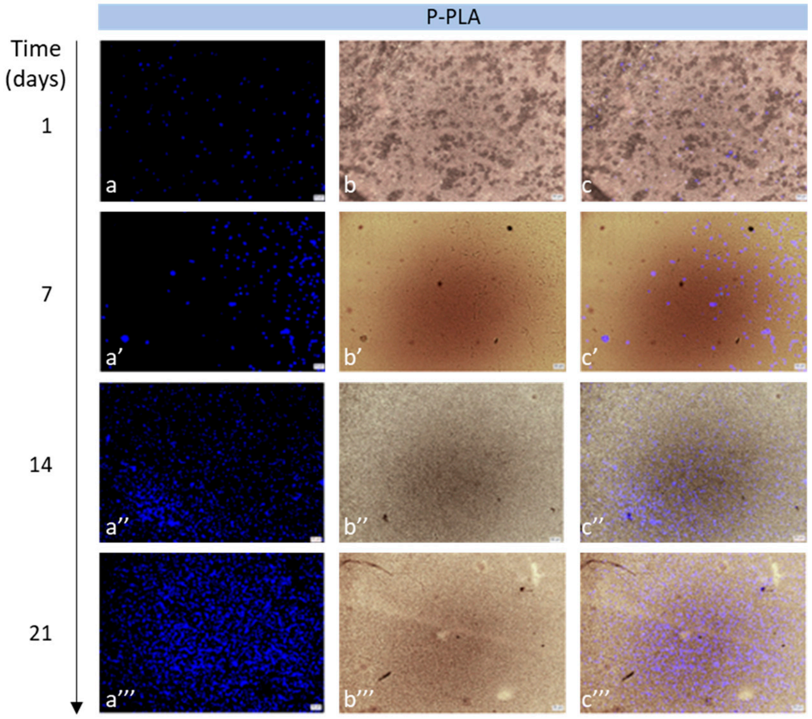

Figure S6. Fluorescence microscopy of MC3T3-E1 cells grown on P-PLA scaffolds for 1, 7, 14 and 21 days. Blue: nuclei, DAPI (a, a', a'', a'''), light (b, b', b'', b''') and merge (c, c', c'', c'''). Magnification 10X.

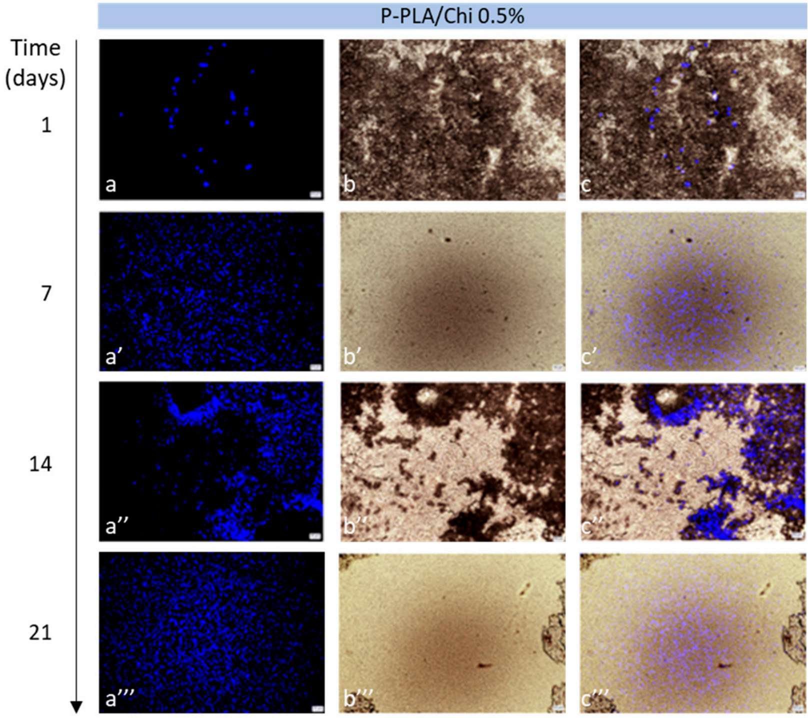

Figure S7. Fluorescence microscopy of MC3T3-E1 cells grown on P-PLA/Chi 0.5% scaffolds for 1, 7, 14 and 21 days. Blue: nuclei, DAPI (a, a', a'', a'''), light (b, b', b'', b''') and merge (c, c', c'', c'''). Magnification 10X.

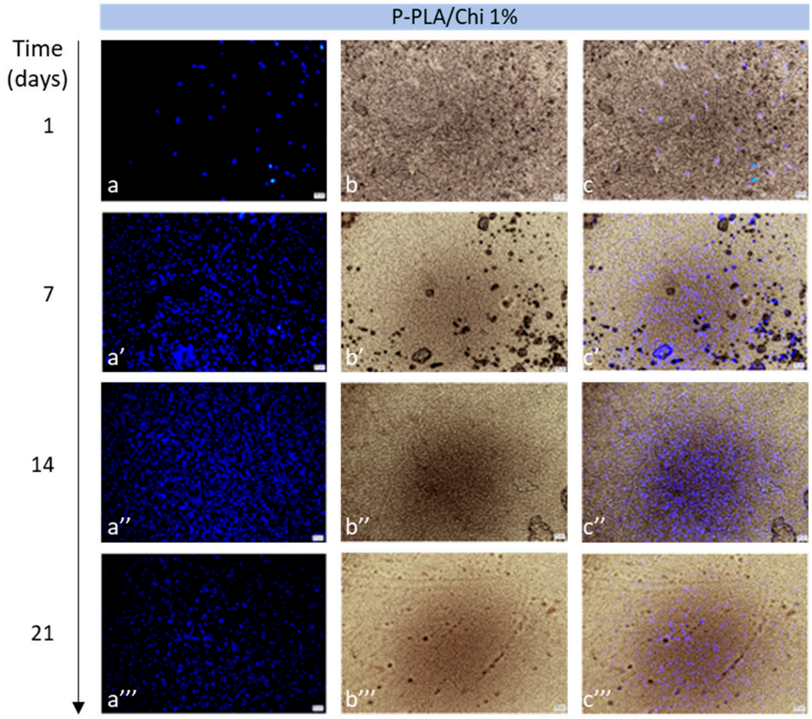

Figure S8. Fluorescence microscopy of MC3T3-E1 cells grown on P-PLA/Chi 1% scaffolds for 1, 7, 14 and 21 days. Blue: nuclei, DAPI (a, a', a'', a'''), light (b, b', b'', b''') and merge (c, c', c'', c'''). Magnification 10X.

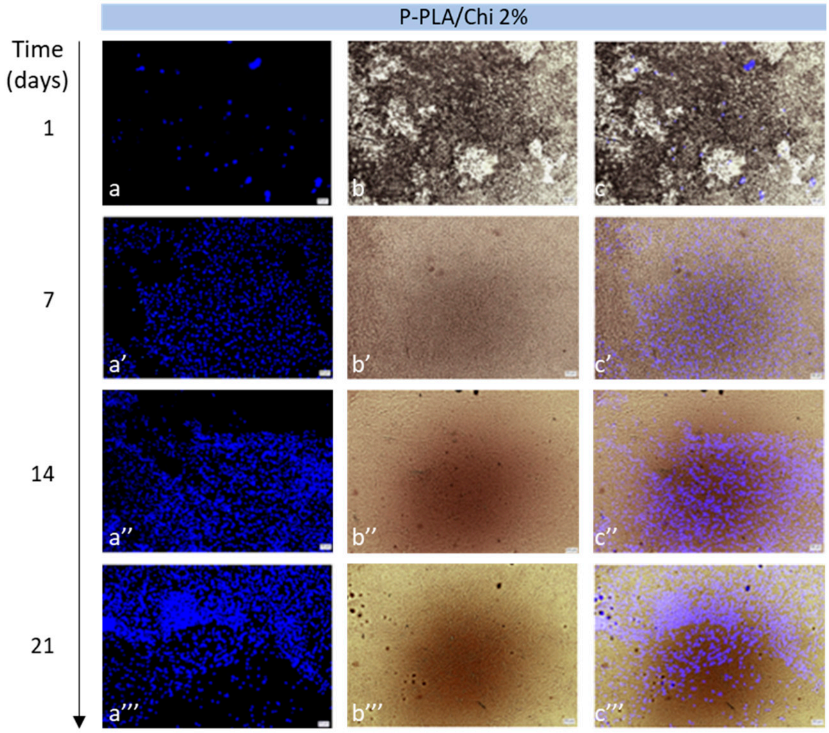

Figure S9. Fluorescence microscopy of MC3T3-E1 cells grown on P-PLA/Chi 2% scaffolds for 1, 7, 14 and 21 days. Blue: nuclei, DAPI (a, a', a'', a'''), light (b, b', b'', b''') and merge (c, c', c'', c'''). Magnification 10X.
